# Novel 1,2,4-Oxadiazole Derivatives in Drug Discovery

**DOI:** 10.3390/ph13060111

**Published:** 2020-05-29

**Authors:** Karol Biernacki, Mateusz Daśko, Olga Ciupak, Konrad Kubiński, Janusz Rachon, Sebastian Demkowicz

**Affiliations:** 1 Department of Organic Chemistry, Faculty of Chemistry, Gdansk University of Technology, Narutowicza 11/12, 80-233 Gdansk, Poland; karbier1@gmail.com (K.B.); olgaciupak@gmail.com (O.C.); januszrachon@gmail.com (J.R.); 2 Department of Inorganic Chemistry, Faculty of Chemistry, Gdansk University of Technology, Narutowicza 11/12, 80-233 Gdansk, Poland; mateusz.dasko@pg.edu.pl; 3 Department of Molecular Biology, Faculty of Biotechnology and Environment Sciences, The John Paul II Catholic University of Lublin, Konstantynów 1i, 20-708 Lublin, Poland; kubin@kul.lublin.pl

**Keywords:** 1,2,4-oxadiazole, synthetic methods, drug design, drug discovery, structure-activity relationship, medicinal application

## Abstract

Five-membered 1,2,4-oxadiazole heterocyclic ring has received considerable attention because of its unique bioisosteric properties and an unusually wide spectrum of biological activities. Thus, it is a perfect framework for the novel drug development. After a century since the 1,2,4-oxadiazole have been discovered, the uncommon potential attracted medicinal chemists’ attention, leading to the discovery of a few presently accessible drugs containing 1,2,4-oxadiazole unit. It is worth noting that the interest in a 1,2,4-oxadiazoles’ biological application has been doubled in the last fifteen years. Herein, after a concise historical introduction, we present a comprehensive overview of the recent achievements in the synthesis of 1,2,4-oxadiazole-based compounds and the major advances in their biological applications in the period of the last five years as well as brief remarks on prospects for further development.

## 1. Introduction

**Figure 1:**
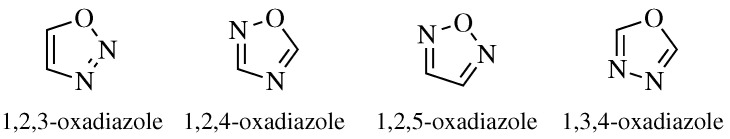
Chemical structures of oxadiazole isomers.

Oxadiazoles are five-membered heterocyclic compounds containing one oxygen and two nitrogen atoms (historically, they were also known as furadiazoles). Depending on the position of nitrogen atoms, oxadiazoles may occur in the form of four different isomers: 1,2,3-oxadiazole, 1,2,4-oxadiazole, 1,2,5-oxadiazole and 1,3,4-oxadiazole (Figure 1). Amongst the isomers, the greatest interest is involved with 1,3,4-oxadiazoles. Their high importance is highlighted by a large number of applications in various scientific areas, e.g., pharmaceutical industry, drug discovery, scintillating materials as well as dyestuff industry [1]. It is also worth noting that compounds containing 1,3,4-oxadiazole unit exhibit a wide range of biological activities such as anticancer, antiparasitic, antifungal, antibacterial, antidepressant, anti-tubercular and anti-inflammatory [2,3,4,5].

**Figure 2:**
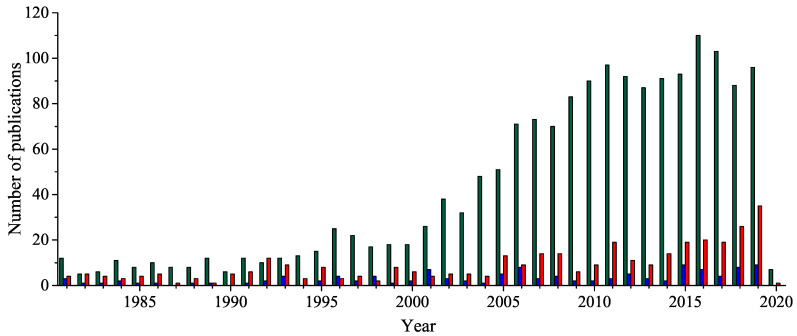
Number of publications containing the keywords: “1,2,4-oxadiazole” (red), “1,2,5-oxadiazole” (blue) and “1,3,4-oxadiazole” (green) in their title since 1980 [6].

According to the *Web of Science* data the scientific attention of 1,3,4-oxadiazoles application is continuously rising since the year 2000 (Figure 2) [6]. On the other hand, 1,2,5-oxadiazole derivatives found application mainly as High Energy Density Materials (HEDMs) as well as biologically active compounds with cytotoxic properties [7,8,9]. Due to the instability and ring-opening of 1,2,3-oxadiazole heterocycle, resulting in substituted diazomethanes formation, this isomer of oxadiazole is least of all explored [10].

## 2. Historical Remarks—1,2,4-Oxadiazole

**Figure 3:**
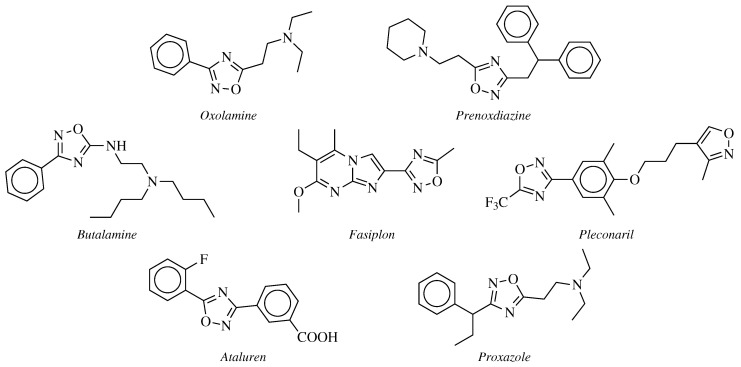
Chemical structures of commercial drugs based on a 1,2,4-oxadiazole scaffold.

The 1,2,4-oxadiazole heterocycle was synthesized for the very first time in 1884 by Tiemann and Krüger and was originally classified as azoxime or furo[*ab*1]diazole [11]. The heterocycle finally caught the attention of chemists almost 80 years after its discovery when photochemical rearrangement of it to the other heterocyclic systems was noted [12,13]. Biological activity studies of 1,2,4-oxadiazole derivatives started in the early 1940s and 20 years later First-In-Class commercial drug containing 1,2,4-oxadiazole ring—*Oxolamine* (Figure 3)—was described and introduced to the pharmaceutical market as a cough suppressant [14,15,16].

**Figure 4:**
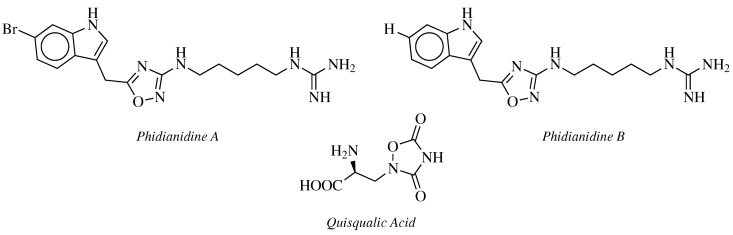
Chemical structures of naturally occurring 1,2,4-oxadiazole-containing compounds.

In the last 40 years, 1,2,4-oxadiazole heterocycle has been widely explored bringing a vast number of compounds exhibiting diverse biological activities such as anticancer, anti-inflammatory, anticonvulsant, antiviral, antibacterial, antifungal, antidepressant, antiangiogenic, analgesic, anti-insomnia, anti-oedema, antiparasitic, and anti-Alzheimer. It was proved that they also show inhibitory potency against Human Deacetylase Sirtuin 2 (HDSirt2), Carbonic Anhydrase (CA), Histone Deacetylase (HDAC), Rearranged during Transfection (RET) kinase, Penicillin-Binding Protein (PBP2a), efflux pump, cyclooxygenases (COX-1 and COX-2) and butyrylcholinesterase (BChE) as well as affinity to σ_1_, σ_2_, orexin, kappa opioid (KOR) and estradiol (ER) receptors (see sections below). Furthermore, 1,2,4-oxadiazole derivatives also found application as supramolecular liquid crystals and HEDMs [7,17,18,19]. Importantly, the heterocycle demonstrates bioisosteric equivalence with ester and amide moieties due to the possibility of creation specific interaction (e.g., hydrogen bonding). It is a particularly useful alternative when the instability of those groups is observed (e.g., when the hydrolysis may appear) [20,21]. Nowadays, there are a few commercially available drugs containing 1,2,4-oxadiazole nucleus such as *Oxolamine*, *Prenoxdiazine* (cough suppressant, Figure 3) *Butalamine* (vasodilator, Figure 3), *Fasiplon* (nonbenzodiazepine anxiolytic drug, Figure 3), *Pleconaril* (antiviral drug, Figure 3), *Ataluren* (Duchenne muscular dystrophy treatment drug, Figure 3) and *Proxazole* (a drug used for functional gastrointestinal disorders, Figure 3) [22,23,24]. It is worth noting that 1,2,4-oxadiazole ring, as the only one of all oxadiazole isomers, occurs in the structures of natural products. For example, in 2011 Carbone M. et al. isolated two indole alkaloids *Phidianidine A* and *Phidianidine B* (Figure 4) from sea slug Opisthobranch *Phidiana militaris* [25].

It was revealed that both Phidianidines exhibit in vitro cytotoxic activity against tumor and non-tumor mammalian cell lines (rat glial—C6, human cervical—HeLa, colon adenocarcinoma—CaCo-2, mouse embryo—3T3-L1 and rat heart myoblast—H9c2) as well as selective agonist properties against protein-tyrosine phosphatase 1B (PTP1B) and chemokine receptor type 4 (CXCR4) [26,27]. *Quisqualic acid* (Figure 4), obtained from seeds of *Quisqualis indica*, is another example of naturally occurring compound bearing 1,2,4-oxadiazole. This alanine-derivative exhibits affinity to metabotropic glutamate receptor type II and IV—attractive molecular targets for the treatment of stroke, epilepsy and neurodegenerative disorders [28,29].

## 3. Methods of 1,2,4-Oxadiazole Synthesis

To date, several methods for synthesis of 1,2,4-oxadiazole derivatives have been developed. Most of them are based on amidoxime and carboxylic acid derivatives heterocyclization or 1,3-dipolar cycloaddition of nitrile and nitrile oxide.

The first approach, proposed by Tiemann and Krüger, uses amidoximes and acyl chlorides and results in the formation of two products (Entry 1, Table 1) [11]. The use of TBAF or pyridine as a catalyst in the aforementioned reaction improves the synthesis efficacy (Entry 2, Table 1) [30]. Reaction between an amidoxime and carboxylic acid esters, particularly methyl and ethyl esters, activated carboxylic acid (with coupling reagents such as EDC, DCC, CDI, TBTU or T3P) or carboxylic acid anhydrides has been also utilized (Entry 3–5, Table 1) [31,32,33,34,35]. Despite the simplicity of the above-described methods, unsatisfactory yields, purification difficulties and inapplicability due to the harsh conditions were usually observed.

It is worth noting that the microwave irradiation (MWI) has been also applied in the heterocyclization of amidoximes and acyl chlorides/carboxylic acid esters in the presence of NH_4_F/Al_2_O_3_ or K_2_CO_3_. This synthetic approach allowed to obtain 3,5-disubstituted-1,2,4-oxadiazoles within extremely short reaction time and with good yields (Entry 6, Table 1) [36,37]. Moreover, a microwave-assisted reaction of aryl-nitrile with hydroxylamine hydrochloride to aryl-amidoxime in the presence of a catalyst (MgO or CH_3_COOH or KF) was also described. This method allowed to obtain 1,2,4-oxadiazoles in a simple two-step procedure (Entry 7, Table 1) [38,39,40]. Interestingly, the application of MWI demonstrated several advantages in comparison with the classical synthetic strategies, e.g., remarkably short reaction time, high yields and simple purification. Furthermore, volumes of volatile organic solvents were highly reduced, which is presently desired and environmentally friendly synthetic approach.

The second method of 1,2,4-oxadiazole formation involves 1,3-dipolar cycloaddition of nitrile oxides and nitriles. Despite the accessibility of starting materials and reagents, this synthetic procedure is usually unfavorable due to the non-reactivity of -CN triple bond and the possibility to formation of 1,2,5-oxadiazole-2-oxides and 1,2,4-oxadiazole-4-oxides through nitrile oxide dimerization [41,42]. However, in 2003 Bokach N. et al. presented a study of 1,3-dipolar cycloaddition of nitrile oxides with nitriles in the presence of platinum(IV) catalyst resulting in the formation of 1,2,4-oxadiazole under mild conditions. However, difficulties such as poor solubility of starting materials, poor yields and expensive catalyst make this synthetic approach still troublesome (Entry 8, Table 1) [43].

**Scheme 1:**
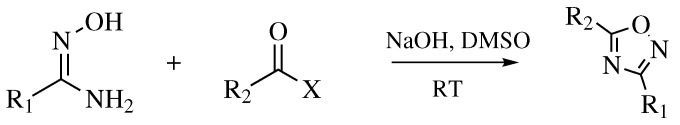
Synthesis of 1,2,4-oxadiazole analogs in the superbase medium (R_1_ = 4-methylphenyl, R_2_ = methyl or phenyl, X = methoxy or ethoxy).

Recently, new synthetic approaches in the formation of 1,2,4-oxadiazoles have been reported. In 2017 Baykov et al. published a study on the first one-pot synthetic procedure for the synthesis of 3,5-disubstituted-1,2,4-oxadiazoles at room temperature (RT) from corresponding amidoximes and carboxylic acids methyl or ethyl esters in the superbase medium NaOH/DMSO (Scheme 1) [44]. This synthetic approach led to obtain diverse oxadiazole analogs isolable via simple purification protocol, although in moderate to long reaction time (4–24h) with poor to excellent yields (11–90%). Moreover, the presence of –OH or –NH_2_ groups in the structure of carboxylic acid ester limited the formation of desired compounds.

**Scheme 2:**
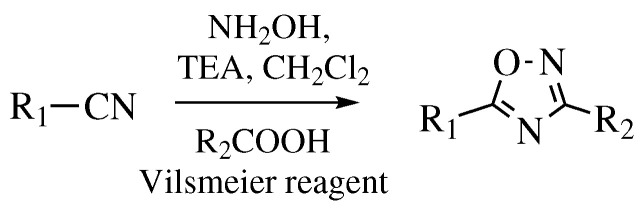
Synthesis of 1,2,4-oxadiazoles with Vilsmeier reagent as a carboxylic acid group activator (R_1_, R_2_ = phenyl, 4-methoxyphenyl, 4-nitrophenyl, 4-chlorophenyl, 2-methoxyphenyl, methyl, 2-thiophenyl and others).

Another interesting, one-pot synthetic procedure of 3,5-disubstituted-1,2,4-oxadiazoles from the corresponding amidoximes and carboxylic acids employing the –COOH group activation via reaction with Vilsmeier reagent (Scheme 2) was reported by Zarei M. [45]. Good to excellent yields (61–93%), a simple purification protocol, an application of readily available starting materials and one-pot synthesis approach highlighted the benefits of using this procedure.

**Scheme 3:**
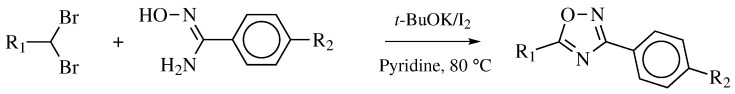
Synthesis of 3,5-substituted-1,2,4-oxadiazoles using *gem*-dibromomethylarenes (R_1_ = phenyl, 4-chlorophenyl, 4-bromophenyl, 4-fluorophenyl, 2-chloro-4-fluorophenyl, 4-cyanophenyl or 4-methoxyphenyl, R_2_ = H, methyl or methoxy).

In 2019, Vinaya K. et al. reported an efficient one-pot synthesis of 3,5-diarylsubstituted-1,2,4-oxadiazoles via a two-component reaction of *gem*-dibromomethylarenes with amidoximes (Scheme 3) [46]. Accessibility of various *gem*-dibromomethylarene derivatives and excellent yields (∼90%) were the main advantages of this method. However, long reaction time and complicated purification protocol diminished its wide application.

**Scheme 4:**
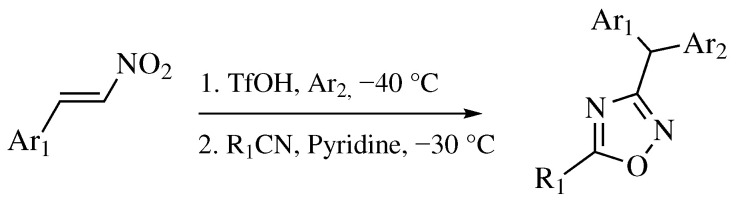
The reaction of nitrostyrenes with arenes and nitriles in the presence of TfOH leading to 1,2,4-oxadiazoles (R_1_ = methyl, ethyl, chloromethyl, phenyl or cyclopropyl, Ar_1_ = phenyl, 4-mehtylphenyl, 4-fluorophenyl, 4-chlorophenyl, 4-bromophenyl, Ar_2_ = phenyl, *p*-ksylene, *o*-ksylene).

Recently, Golushko A. et al. developed a novel synthetic method of 1,2,4-oxadiazoles based on tandem reaction of nitroalkenes with arenes and nitriles in the presence of TfOH (Scheme 4) [47]. Despite the excellent yields (∼90% in most cases) and short reaction time (10 min), the usage of a superacid requires resistant starting materials, which can be a serious limitation.

**Scheme 5:**
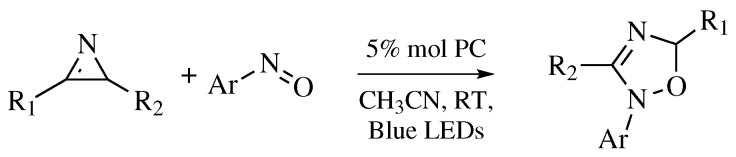
[3+2]-cycloaddition of 2*H*-azirines and nitrosoarenes (R_1_, R_2_, Ar = phenyl, 4-methylphenyl, 4-chlorophenyl, 4-bromophenyl, 2-methylphenyl or 2-pyridinyl).

In 2019, Cai B. et al. presented a study on [3+2]-cycloaddition reaction of disubstituted-2*H*-azirines with nitrosoarenes under irradiation of visible light and in the presence of organic dye photoredox catalyst—9-mesityl-10-methylacridinium perchlorate (PC), resulting in the formation of 2,3,5-trisubstituted-1,2,4-oxadiazoles (Scheme 5) [48]. This synthetic strategy provided a “green chemistry” and efficient synthetic method of 1,2,4-oxadiazole synthesis. Despite promising and environmentally friendly conditions, moderate yields (35–50%) limit the wide application of this type of transformation. However, further studies for improving this rational method are still ongoing.

Despite quite a large number of synthetic methods of 1,2,4-oxadiazoles, low yields, long reaction times, an usage of volatile and toxic organic solvents, purification difficulties and a presence of active groups in the structure of reagents (e.g., –NH_2_, –OH) often limit their application. For that reason, novel, effective and green chemistry-based synthetic methods of 1,2,4-oxadiazoles are still sought-after. Very recently, mechanochemistry—which refers to the reaction in the solid-state induced by the mechanical energy (e.g., grinding or milling)—became much more intensely explored, due to the increased reaction rate and quantitative yields in the absence of solvents (or only in minimal volumes), though none article has been published on the 1,2,4-oxadiazole formation via mechanochemistry [49]. Therefore, we hope that this kind of synthetic approach, due to its advantages, will find application in the efficient and environmentally friendly synthesis of the 1,2,4 oxadiazole based compounds in the near future.

## 4. Anticancer Agents

**Figure 5:**
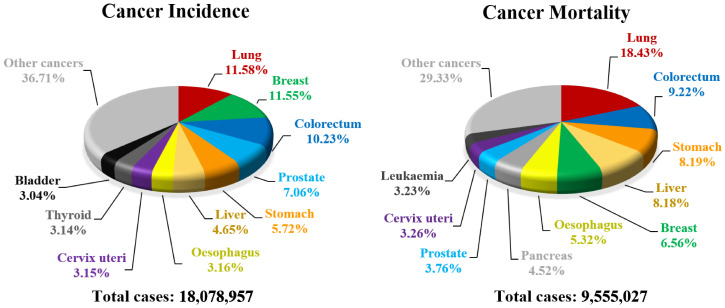
Estimated number of cancer incidences and cancer-related deaths in 2018.

Every year cancer impacts about 20 million people all over the world resulting in deaths counting in millions (Figure 5). Unfortunately, a number of new cancer cases is still rising and almost 30 million people will be diagnosed with carcinoma by 2040 in high-developed countries [50]. For that reason, finding new cancer treatments or effective drugs is one of the greatest needs of the current community and a challenge for modern medicine. Biological evaluation of 1,2,4-oxadiazoles revealed that some of their derivatives are potent anticancer agents. The greatest breakthrough came with the discovery of 3,5-diarylsubstituted derivatives of 1,2,4-oxadiazole as a new series of apoptosis inducers [51]. Since then, exploration of the anticancer activity of 1,2,4-oxadiazole derivatives has been started resulting in a creation of a wide library of compounds [52,53].

**Figure 6:**
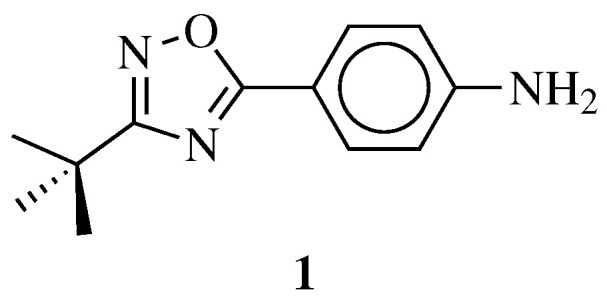
Chemical structure of 4-(3-(*tert*-butyl)-1,2,4-oxadiazol-5-yl)aniline **1**.

Recently, Maftei C. V. et al. reported the synthesis of 4-(3-(*tert*-butyl)-1,2,4-oxadiazol-5-yl)aniline (**1**, Figure 6), which exhibits moderate activity with a mean IC_50_ value of approximately 92.4 μM against panel of 11 cancer cell lines (human colon adenocarcinoma—CXF HT-29, human gastric carcinoma—GXF 251, human lung adenocarcinoma—LXFA 629, human non-small cell lung carcinoma—LXFL 529, breast cancer-derived from athymic mice’ lung metastatic site—MAXF 401, human melanoma—MEXF 462, human ovarian adenocarcinoma—OVXF 899, human pancreatic cancer—PAXF 1657, human pleuramesothelioma cancer—PXF 1752, human renal cancer—RXF 486, human uterus carcinoma—UXF 1138). Importantly, compound **1** became a precursor for synthesis of novel compounds with greater antiproliferative activities [54].

Further modification of **1** led to the discovery of its derivative **2** (Table 2) exhibiting significantly greater antitumor activity evaluated against a panel of 12 human tumor cell lines (CXF HT-29, GXF 251, LXFA 629, LXFL 529, MAXF 401, MEXF 462, OVXF 899, PAXF 1657, human prostate cancer—PRXF 22Rv1, PXF 1752, RXF 486 and UXF 1138), especially toward OVXF 899 and PXF 1752 cell lines with the IC_50_ values of 2.76 and 9.27 μM, respectively. Moreover, compound **2** showed high selectivity against renal cancer cell line with the IC_50_ = 1.143 μM [55]. In addition, the same research team reported new gold(I) complexes with 1,2,4-oxadiazole-containing *N*-heterocyclic carbene ligands. Obtained results clearly revealed impressive potency of imidazolium salts. The most active derivative **3** (Table 2) showed extremely low IC_50_ values from 0.003 to 0.595 μM against the same panel of 12 cancer cell lines with highest activity in an in vitro assays with LXFA 629 and MAXF 401 cells (IC_50_ = 0.003 μM for both of them) [56]. Thus, **3** seems to be an ideal candidate for further evaluation. More advanced in vivo studies may reveal some additional features, although no information has been published up to date.

**Figure 7:**
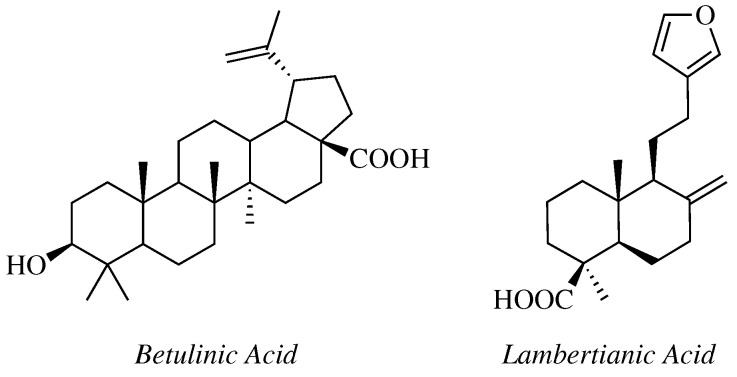
Chemical structure of *Betulinic Acid* and *Lambertianic Acid*.

In a study reported by Challa K., Krishna C. and coworkers C28-modified *Betulinic Acid* (Figure 7) bearing 1,2,4-oxadiazole ring connected via ester or amide linker have been synthesized and evaluated against human colon carcinoma (Colo 205), human liver cancer (Hep G2) and HeLa cell lines [57,58]. Performed screening revealed moderate potential of all obtained derivatives with the highest biological activities for analogs **4a–4d** (Table 2) (the IC_50_ values in a range of 26.1–34.3 μM). However, the obtained compounds turned out to be still weaker than reference compound—etoposide (the IC_50_ values of 0.42–22.5 μM), a topoisomerase II enzyme inhibitor, which is currently used as medication in the treatment of cancer diseases (e.g., lung, ovarian, testicular cancers, leukemia, neuroblastoma and lymphoma) [59]. Interestingly, the impact on the biological activity of compounds by switching the ester moiety with amide was negligible.

Mironov et al. carried out a synthesis of several derivatives of *Lambertianic acid* (Figure 7) by the introduction of substituted-1,2,4-oxadiazole heterocycle at the C16 position [60]. Obtained compounds were tested in comparison with doxorubicin—widely used anticancer agent in the treatment of breast, bladder carcinomas, lymphoma, and acute lymphocytic leukemia [61,62]. Obtained outcomes by Mironov et al. revealed that **5a**–**b** (Table 2) exhibited more favorable biological activity than *Lambertianic Acid* itself with the GI_50_ values at sub-micromolar concentration against human childhood and adult T acute lymphoblastic leukemia (CEM-13), MT-4, and human adult acute monocytic leukemia (U-937) cancer cell lines. It is worth noting that **5a**–**b** demonstrated greater cytotoxic activity than doxorubicin. Additional biological studies indicated that activities of **5a**–**b** against human breast adenocarcinoma—MCF-7, MDA-MB-231 and human melanoma—MEL-8 cancer cell lines were slightly lower than the reference compound. Interestingly, flow cytometry assay revealed that the above-mentioned compounds are potent inducers of apoptosis in MCF-7, MDA-MB-231 and MEL-8 cell lines and are acting in a dose-dependent manner.

In the study of Kucukoglu K. et al. a series of Schiff bases fused with 1,2,4-oxadiazole heterocycle has been synthesized and evaluated in vitro against a panel of 8 cancer cell lines [63]. Results revealed that **6a**–**c** (Table 2) exhibited higher biological potency (CC_50_ = 137.3, 79.0 and 140.3 μM, respectively) against Ca9-22 cell line than 5-fluorouracil (a multi-acting agent used in the treatment of colon, esophageal, stomach, breast and pancreatic cancers) applied as a reference (CC_50_ = 214.3 μM). On the other hand, the cytotoxic potency of obtained compounds occurred to be far weaker than doxorubicin. For this reason, modifications of chemical structure including a different substitution of terminal aromatic rings or an introduction of additional pharmacophores are worth of consideration to improve biological activity.

Moniot S., Forgione M. et al. reported a study of about 40 novel substituted 3-aryl-5-alkyl-1,2,4-oxadiazole derivatives as selective inhibitors of HDSirt2—NAD^+^ lysine deacetylase—an attractive target for treating neurodegenerative disorders, metabolic dysfunctions, age-related diseases and cancer [64]. The biological activity of obtained derivatives was assessed in a continuous assay using an α-tubulin-acetylLys40 peptide as a substrate. Based on the detailed structure-activity relationship (SAR) studies, compounds **7a** and **7b** (Table 2) emerged as the most potent HDSirt2 inhibitors when tested against human leukemia cell lines (U-937, NB4, HL-60, and K562) and MDA-MB-231 cell line. Analog **7a** was able to induce apoptotic death in over 80% of NB4, K562 and MDA-MB-231 cancer cell at the concentration of 25 μM. Moreover, **7b** achieved the same effect at 10 μM. According to the western blot analyses, the involvement of HDSirt2 inhibition for apoptotic death induction has been confirmed. In addition, the crystal structure of 1,2,4-oxadiazole derivatives in complex with HDSirt2 revealed yet unexplored subcavity, which may be extremely useful for further inhibitors development [64].

In 2017, Avanzo R. E. and coworkers synthesized 9 novel diheterocyclic-ribose fused derivatives containing 5-substituted-1,2,4-oxadiazole framework. Their previous study suggested that 5-deoxy-5-*S*-(1,2,4-triazol-3-yl)-2,3-*O*-cyclopentylidene-β-D-ribofuranoside derivatives are moderate antitumor agents. It turned out that the introduction of 5-substituted-1,2,4-oxadiazole heterocycle into the ribose-derivative structure improved anticancer activity [65,81]. Obtained compounds were tested against human lung (A549), SW1573, HeLa, human breast (HBL-100), T-47D, and human colon (WiDr) cancer cell lines. Among them, compound **8** (Table 2) showed the highest antiproliferative potency and selectivity against WiDr with the GI_50_ value of 4.5 μM. It was noticed that the presence of electron withdrawing group (EWG) at the *para* position of the aromatic ring occurred to be crucial to ensure high biological activity.

**Figure 8:**
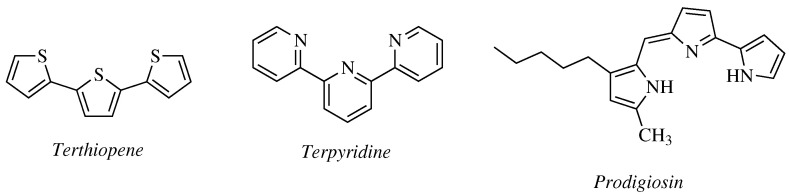
Chemical structure of *Terthiopene*, *Terpyridine*, and *Prodigiosin*.

Recently, Abd el hameid M. K. reported 15 novel 1,2,4-oxadiazole derivatives as analogs of *Terthiopene*, *Terpyridine*, and *Prodigiosin* (Figure 8)—naturally occurring compounds with potent cytotoxic and pro-apoptotic properties against various types of carcinoma [66]. Obtained compounds were preliminary evaluated against MCF-7 cancer cell line and the most potent were selected for further evaluation toward human colon cancer—HCT-116 cell line. Obtained results revealed that **9a**–**c** (Table 2) exhibited the highest activity with the IC_50_ values of 0.48, 0.78, 0.19 μM and 5.13, 1.54, 1.17 μM against MCF-7 and HCT-116, respectively. In addition, their biological activities were comparable or greater than reference *Prodigiosin* (the IC_50_ values of 1.93 and 2.84 μM against MCF-7 and HCT-116 cell line, respectively). Interestingly, flow cytometry analysis revealed that the above-mentioned compounds were able to arrest cell proliferation at G1 phase in MCF-7 cells and were triggering apoptosis via increasing of caspase3/7 activity, thus are suitable for further development as potent anticancer agents.

In 2018, de Oliveira V. N. M. and collaborators synthesized a series of substituted *N*-cyclohexyl-3-aryl-1,2,4-oxadiazole-5-amines from corresponding arylamidoximes and DCC under MWI and determined their antitumor activity against HCT-116, human prostate (PC-3) and human astrocytoma (SNB-19) cancer cell lines [67]. Compounds **10a** and **10b** (Table 2) exhibited the highest activity and were further evaluated against five cell lines—HCT-116, PC-3, SNB-19, mouse melanoma (B16F10) and mouse adipose (L929). Their activity expressed by the IC_50_ values ranged from 13.6 to 48.37 μM, nonetheless, the levels of inhibition were still far from reference compound—doxorubicin, thus additional modifications of a chemical structure are required for improvement of the activity.

Kumar P. S. et al. synthesized a novel series of *bis*-1,2,4-oxadiazole-fused-benzothiazole derivatives and examined their biological activity against A549, MCF-7, human amelanotic melanoma (A375) and HT-29 cancer cell lines [68]. Most of the obtained analogs exhibited moderate activity except for **11a** and **11b** (Table 2), which demonstrated comparable or slightly lower potency than combretastatin-A4 (reference compound, which is phosphate-based anticancer drug used in the treatment of many carcinomas, as microtubule destabilizing agent, designed to interrupt the blood vessels formation in cancer tissue and cause central necrosis). Analog **11a** showed the highest activity toward A549 cell line with the IC_50_ value of 0.11 μM, although **11b** turned out to exhibit higher activity against MCF-7, A375 and HT-29 cell lines (the IC_50_ values of 0.2, 2.09 and 0.76 μM, respectively). In addition, the SAR studies revealed that the presence of electron donating groups (EDG) greatly improved activity. In comparison, the introduction of EWG was related with decreasing of antiproliferative potency.

In the study of Pervaram S. a synthesis and biological evaluation of 10 new compounds based on 1,2,4-oxadiazole derivatives containing benzofuran group have been carried out. Antiproliferative potency of obtained compounds was estimated in MTT assay against MCF-7, A375, and HT-29 cancer cell lines. Compounds **12a**–**d** (Table 2) showed promising cytotoxic activity at sub-micromolar concentrations (comparable or higher than the reference compound—combretastatin-A4). Interestingly, the replacement of EDG or EWG with halogen atoms in the phenyl ring was related with drastic decrease of biological activity [69].

In 2018, Chakrapani B. et al. published an article regarding the synthesis and examination of cytotoxic activity of 1,2,4-oxadiazole-fused-imidazothiadiazole derivatives against human cancer cell lines (A375, MCF-7, and ACHN), where doxorubicin has been used as a reference compound. Two of the obtained compounds **13a**–**b** (Table 2) showed good antitumor activity with the IC_50_ values between 0.11–1.47 μM against the aforementioned cancer cell lines. Interestingly, the reference compound exhibited similar or slightly lower anticancer activity (the IC_50_ values in the range of 0.79 to 5.51 μM) [70].

Srinivas M. et al. synthesized a series of 1,2,4-oxadiazoles linked with benzimidazole derivatives and evaluated their antitumor activities against MCF-7, A549, A375 cancer cell lines [71]. Compounds **14a**–**d** (Table 2) exhibited higher biological activity than doxorubicin with the IC_50_ values in a range of 0.12–2.78 μM against MCF-7, A549, A375. In addition, the replacement of EDG or EWG with halogen atoms in the phenyl ring decreased the antiproliferative activities of tested compounds. Further evaluation of 1,2,4-oxadiazole-benzimidazole derivatives based on comprehensive in vivo studies should reveal their clinical potential, however, none article has been published in this area up to date.

**Figure 9:**
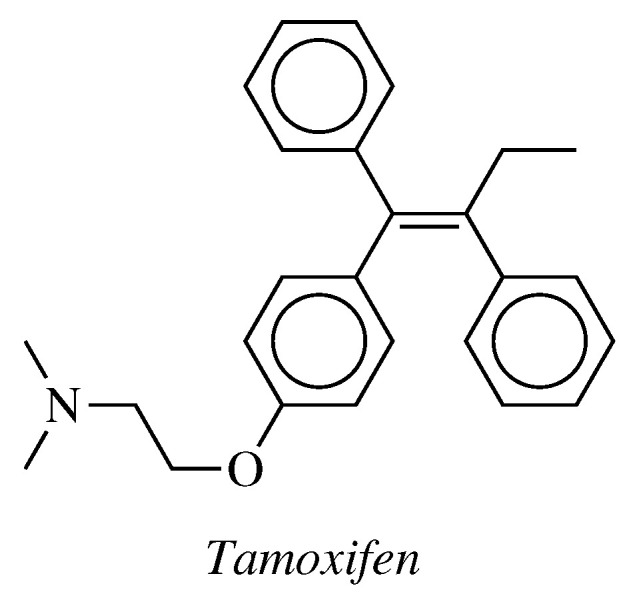
Chemical structure of *Tamoxifen*.

*Tamoxifen* (Figure 9) is sold under the brand name Nolvadex, Tamifen, Genox, and many others. It is a drug used as a Selective Estrogen-Receptor Modulator (SERM) and has been applied in early hormone-dependent breast cancer treatment and prevention for over 40 years [82]. Unfortunately, long-term treatment with SERMs often led to many undesirable side effects such as blood clots, strokes, cataracts, bone loss, mood swings, depression, risk of heart attack and failure, loss of libido and high probability of cancer recurrence or even formation of new ones—endometrial and uterine [83,84,85,86]. For that reason, further development of *Tamoxifen* derivatives are still of special significance.

A new series of 3,4-diaryl-1,2,4-oxadiazolidin-5-ones have been synthesized as analogs of *Tamoxifen* and their biological potential and ability to activate apoptosis were determined in vitro against MCF-7 cancer cell line [72]. Received outcomes showed that compound **15** (Table 2) exhibited the highest cytotoxic effect against MCF-7 cell line with the IC_50_ value of 15.63 μM, which was similar to that of *Tamoxifen*—reference compound (the IC_50_ value of 10.38 μM). Western blot analysis revealed that the above-described compound increased p53 expression level and caspase-3 cleavage in MCF-7 cells leading to activation of the apoptotic death. Additionally, molecular docking studies using the crystal structure of ER suggested strong hydrophobic interactions between aromatic rings of 1,2,4-oxadiazolidin-5-ones and amino-acid residues of the receptor, which were similar to those that *Tamoxifen* creates. Despite promising activities of presented derivatives, chemical structure modification is still required to find new *Tamoxifen* analogs based on 1,2,4-oxadiazole core with better activity and pharmacological profile.

In the recent study performed by Krasavin M. et al. a novel series of substituted 1,2,4-oxadiazole-arylsulfonamides has been discovered as selective CA inhibitors with potential application in the cancer therapies [73,74]. An extensive research (conducted by the same research group) exploring various substituted heterocyclic compounds (including 1,3-oxazole, isoxazole, imidazoline and pyrazole) with sulfonamide moiety indicated that 1,2,4-oxadiazol-5-yl-benzene sulfonamides were able to demonstrate extremely high biological activity and selectivity [87,88,89,90]. Inhibitory potency of synthesized compounds was measured with the use of CO_2_ hydration stopped-flow biochemical assay against two cytosolic Human Carbonic Anhydrases (*h*CA I and II) and two membrane-bound cancer related (*h*CA IX and XII) CA. It turned out that fourteen out of sixty obtained compounds were able to selectively inhibit *h*CA at nanomolar, sub-nanomolar and even picomolar concentrations range [73,74]. The most active compound **16a** (Table 2) showed K_i_ values of 89 pM (*h*CA IX) and 0.75 nM (*h*CA II). Further in vitro evaluation of 1,2,4-oxadiazol-5-yl sulfonamides against non-cancerous human retinal pigment epithelial cell line (ARPE-19) and cancerous cell lines (pancreas ductal adenocarcinoma—PANC-1 and melanoma—SK-MEL-2 cell line) under normoxic and hypoxic conditions indicated that **16a** and **16b** (Table 2) were the most promising compounds. Analog **16a** showed the highest selectivity and activity against SK-MEL-2, while **16b** was the most effective toward PANC-1. Further exploration of 1,2,4-oxadiazol-5-yl benzene sulfonamides may lead to the discovery of potent small-molecule membrane-bound CA inhibitors as a therapeutic intervention in cancer.

New analogs of nortopsentin—a marine natural product—in which 1,2,4-oxadiazole framework replaced the central imidazole heterocycle have been synthesized and examined against HCT-116 cancer cell line [75]. Compounds **17a** and **17b** (Table 2) showed the highest cytotoxic activity reaching the IC_50_ values in the micromolar range. Further in vitro evaluation against MCF-7, HeLa and CaCo-2 cancer cell lines were performed. Analogs **17a** and **17b** exhibited the highest biological activity toward MCF-7 with the IC_50_ values of 0.65 and 2.41 μM, respectively. On the other hand, the rest of synthesized nortopsentin derivatives showed approximately 100 times lower anticancer activity. Additionally, flow cytometry analysis revealed that **17a** and **17b** were able to arrest cell cycle at G0-G1 phase. The exact mechanism of drug’s activity is still unknown, however, it is potentially related to the disruption of the cell machinery promoting DNA duplication. Surprisingly, the above-mentioned compounds did not affect the viability of normal-like cells at 10 μM. Therefore, further development of nortopsentin analogs containing 1,2,4-oxadiazole ring may lead to the discovery of new small-molecule anticancer agent. Due to the moderate antitumor activity their structural modification is still necessary.

Recently, Polothi R. et al. published an article about 1,2,4-oxadiazole-1,3,4-oxadiazole-fused derivatives synthesis and their biological evaluation against MCF-7, A549 and MDA MB-231 cancer cell lines [76]. Obtained compounds showed from moderate to excellent anticancer potency. The most active derivatives **18a–c** (Table 2) exhibited the IC_50_ values at sub-micromolar concentration. Obtained results clearly showed that the introduction of EWG in the structure of 5-aryl-1,2,4-oxadiazole aromatic ring caused an increase of antitumor activity. Additionally, the introduction of a nitro group at the *meta* position turned out to be more favorable than *para* substitution. Furthermore, molecular docking studies revealed that compound **18b** is a strong tubulin-binding agent and exhibit a high affinity to target protein epidermal growth factor receptor. It seems that further development of 1,2,4-oxadiazole linked 1,3,4-oxadiazole derivatives may lead to novel, potent anticancer agents.

**Figure 10:**
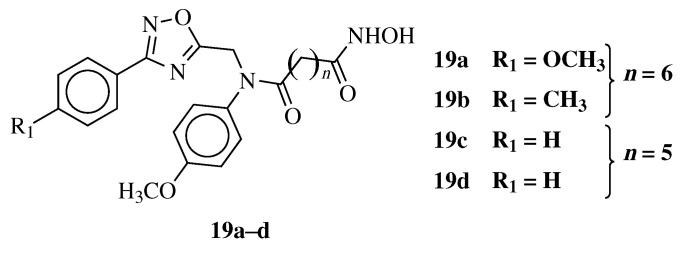
Chemical structures of hydroxamate-based 1,2,4-oxadiazoles.

In the study of Yang F., Shan P. and collaborators a new series of 1,2,4-oxadiazole hydroxamate-based derivatives have been described as HDAC inhibitors [77,78]. Four obtained compounds **19a–d** (Figure 10) were studied against HDAC-1 for evaluation of their inhibitory ability at 20 nM concentration and compared to reference compound—suberanilohydroxamic acid. Suberanilohydroxamic acid (SAHA, also known as Vorinostat) marketed under the name Zolinza, approved by Food and Drug Administration (FDA) agency in 2006 in the treatment of cutaneous T cell lymphoma. It was proved that compounds **19a**–**d** were less active than SAHA and were capable of inhibiting HDAC-1 action only up to 50%. Also, the presence of a five-methylene linker turned out to be more effective than six-methylene, which was the basis for further modification. Intriguingly, the substitution of 1,2,4-oxadiazole heterocycle was crucial to ensure high HDAC-1 inhibitory activity. Despite the minuscule structural difference, it turned out that 3-aryl-5-alkyl-1,2,4-oxadiazole derivatives **20a**–**c** (Table 2) exhibited much higher inhibitory potency than that of 5-aryl-3-alkyl-1,2,4-oxadiazole derivatives (**19a**–**d**) and were capable of stopping HDAC-1 action up to 90% at remarkably low concentration of 20 nM [77].

Additionally, influence of EWGs and EDGs as well as optimal substitution position were investigated. SAR studies revealed that the introduction of a substituent only slightly affected the inhibitory potency. Compounds **20a**–**c** exhibited the most favorable IC_50_ values against HDAC-1 (8.2, 10.5 and 12.1 nM) and slightly higher than that of SAHA (the IC_50_ value of 15.0 nM). Subsequently, the afore-described derivatives were additionally examined in vitro for their anticancer activity toward human hepatocellular (HCCLM3) and HepG2 cancer cell lines. Compounds **20a** and **20b** showed the highest anticancer activity in the micromolar range (comparable to that of SAHA). Furthermore, flow cytometry analysis revealed that **20a** and **20b** were able to greatly induce cell apoptosis. Generally, novel 1,2,4-oxadiazole HDAC inhibitors may be a very promising agent for hepatic carcinoma treatment.

Recently, Yang Z. and coworkers reported a series of HDAC inhibitors containing 1,2,4-oxadiazole heterocycle [79]. Amongst synthesized derivatives, compound **21** (Table 2) showed the most potent HDAC inhibitory activity, particularly against HDAC-1, -2 and -3, with the IC_50_ values of 1.8, 3.6 and 3.0 nM, respectively. Detailed SAR studies revealed that the presence of the linker between hydroxamic acid moiety and pyrimidine heterocycle (e.g., methylene, ethylene, vinyl linker) as well as shifting or replacement of *p*-methyl group in the structure of terminal aromatic ring were responsible for decreasing of the inhibitory potency. Moreover, in vitro studies against a panel of 12 cancer cell line (colon, ovarian, breast, liver, myeloma, lymphoma) for compound **21** showed its extremely high activity against all of the evaluated cancer cell lines with the IC_50_ values in a range from 9.8 to 44.9 nM (in comparison, the IC_50_ values for SAHA were determined between 0.514–5.541 μM). In addition, the antiproliferative activity of **21** was also evaluated against primary Acute Myeloid Leukemia (AML) cell line derived from three diverse patients (the IC_50_ values of 22.2–77.4 nM). Yang Z. et al. performed in vivo studies based on Burkitt’s lymphoma Daudi xenograft model and showed that **21** was able to remarkably reduce tumor growth, up to 53.8% when administered orally at 20 mg/kg with no significant side effects. The research group of Yang Z. led to the discovery of extremely potent HDAC inhibitors as anticancer agents, perfectly suitable for further clinical studies.

**Figure 11:**
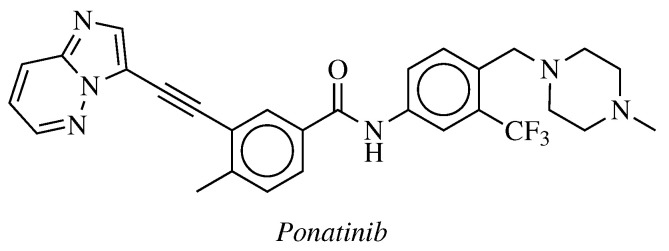
Chemical structure of *Ponatinib*.

Han M. et al. synthesized a novel class of compounds as analogs of *Ponatinib* (Figure 11) [80], which is a multi-targeted tyrosine-kinase inhibitor used in the treatment of chronic myelogenous leukemia (in 2013 its clinical application has been suspended, due to the life-threatening blood clots, and many other adverse effects, including hypertension, headache, fatigue, abdominal pain, dry skin and many more) [91,92]. The applied strategy was based on the replacement of the alkynyl linker between imidazopirydazine and benzamide moiety present in the *Ponatinib* structure with different five-membered heterocycle rings—1,3,4-oxadiazole, 1,2,4-oxadiazole, and oxazole. In the course of the investigation, 1,2,4-oxadiazole-Ponatinib analogs exhibited the highest activity in enzyme-linked immunosorbent assay (ELISA). Further SAR analysis revealed that the presence of chlorine atom attached to the benzamide aromatic ring is crucial for high RET inhibitory activity and its replacement diminished the activity. Compound **22** (Table 2) inhibited RET enzyme in an ELISA assay with the IC_50_ value of 7.3 nM. Additionally, western blot analysis proved that **22** was able to greatly block the RET signaling pathway and showed similar potency to *Ponatinib* against the proliferation of gatekeeper mutant V804 M-driven cell with an IC_50_ value of 441.8 nM. Summarizing, alkynyl linker replacement with 1,2,4-oxadiazole heterocycle enhanced the biological activity of derivatives against the RET enzyme, thus their further development may lead to the discovery of novel Ponatinib-like drugs with no adverse effects.

## 5. Antimicrobial Agents

So far literature have listed over 1400 different species of microbials (including bacteria, viruses, protozoa, fungi and helminthes) able to elicit illnesses in human body which very often leads to death. Surprisingly, only 20 of them (mainly bacteria) are responsible for approximately two thirds of the fatal cases [93]. Estimated deaths from infections is continuously falling, from 16 million in 1990 to approximately 15 million to forecasting 13 million in 2050 in high-developed countries. However, people are still suffering an enormous burden dint of pneumonia, HIV/AIDS, tuberculosis, malaria, diarrhea and many other diseases [94,95]. In light of the numerous pandemic threats in European countries and the world, including the recent infections with the SARS-CoV-2 virus causing COVID-19, discovering new, effective antibacterial/antiviral drugs and the development of modern therapies are two challenges of paramount importance.

In 2014 O’Daniel P. I., Mobashery S., and Chang M. et al. from the University of Notre Dame in the United States put a great effort into the development of 1,2,4-oxadiazole as new antibiotics and discovered a new class of non-β-lactam drugs that were able to inhibit PBP2a of Methicillin-Resistant *Staphylococcus aureus* (MRSA) [96]. Detailed computer screening allowed to select 29 compounds from 1.2 million compounds (ZINC database), which were tested for their antibacterial activity against ESKAPE pathogens and agent **23** (Table 3) emerged as the most promising. Its further evaluation brought an enormous number of derivatives and led to the discovery of **24** (Table 3), which exhibited superior antibacterial activity against Vancomycin-Resistant *S*. *aureus* (VRSA), Vancomycin-Resistant *Enterococcus faecium* (VRE) as well as MRSA with the MIC values ranging from 1 to 2 μg/mL. Moreover, rapid-time kill kinetics studies revealed that **24** was able to cause instant cell death of VRE and Daptomycin-non-Susceptible isolates at 4 mg/L in 1 h resulting in better outcomes than reference compound—daptomycin [97]. Further modifications of **24** and very detailed SAR analysis allowed to obtain a wide library of its analogs (counting in hundreds of derivatives) and resulting in discovery of 5-(1*H*-indol-5-yl)-3-(4-(4-(trifluoromethyl)phenoxy)phenyl)-1,2,4-oxadiazole (also called as **ND-421**, Table 3). **ND-421** exhibited longer half-time, a high volume of distribution, low clearance, excellent bioavailability, 3 times longer postantibiotic effect than linezolid without inoculum effect with unaltered biological activity [98,99,100]. Additionally, in vitro studies against *S*. *aureus*, which exhibits two- and four-fold increased resistance, revealed first-time-reported, unique resistance mechanism to 1,2,4-oxadiazoles in MRSA. Moreover, those pathogen mutants did not show increased resistance to ampicillin, imipenem, linezolid, and vancomycin antibiotics (which are last drug-based defense against MRSA and VRSA) which made **ND-421** a perfect alternative drug for refractory microorganisms [101]. It is also worth pointing out that **ND-421** showed high synergy with other β-lactams (oxacillin, piperacillin, imipenem, meropenem and cefepime) unlike to non-β-lactam antibiotics (vancomycin, linezolid, gentamicin, doxycycline and azithromycin). Recently, the same research team performed additional in vitro studies of **ND-421** against 210 different MRSA and VRE, which exhibited the MIC_50_ values of 4 μg/mL in all examined strains. Moreover value of MIC_50_ were consistently lowered when studied compound was used in combination with oxacillin [102,103]. In summary, 1,2,4-oxadiazoles **23**, **24** and **ND-421** are extremely potent and very promising non-β-lactam bactericidal antibiotics against Gram-positive multi-resistant bacteria suitable for further in vivo evaluation and clinical studies, although no information has been published up to date.

In the recent study of Krolenko K. et al. a new series of 5-(1*H*-1,2,3-triazol-4-yl)-1,2,4-oxadiazole derivatives as antimicrobial agents have been synthesized and examined by agar diffusion test against Gram-positive (*S*. *aureus*, *B*. *subtilis*, *E*. *coli*) and Gram-negative bacteria (*P*. *vulgaris*, *P*. *aeruginosa*) as well as fungi (*C*. *albicans*). Amongst three different series, compound **25** (Table 3) exhibited the highest biological activity with grow inhibition zone in a range of 20–25 mm, better than reference compounds—metronidazole and syntomycin—commonly used antibiotics (grow inhibition zone of 14–17 mm) [104]. Despite the high potential of 5-(1*H*-1,2,3-triazol-4-yl)-1,2,4-oxadiazole derivatives, no further work has been published.

In 2018 Cunha F. S. et al. synthesized a series of 3,5-diarylsubstitued-1,2,4-oxadiazole derivatives and determined their biological activity against *E*. *coli*, *P*. *aeruginosa*, *E*. *faecalis*, *P*. *mirabilis* and *S*. *aureus* using agar diffusion method [105]. Received results showed that some of the obtained compounds were able to inhibit *P*. *mirabilis*, *E*. *faecalis* and *E*. *coli* growth, however, activities against *S*. *aureus* and *P*. *aeruginosa* were not observed. Derivative **26** (Table 3) was the most potent with the MIC value of 60 μM against *E*. *coli*. Additionally, the replacement of nitro group or chlorine atom attached to aromatic rings diminished antimicrobial activity. It turned out that the presence of a nitro group is crucial for the activity, because it promotes the formation of radicals via bioreduction, which leads to peroxidation of proteins and biological membranes or inhibition of crucial enzymes [113].

In 2016, Shi G. et al. reported a synthesis and biological evaluation of 3′,4′-diaryl-4′*H*-spiro[indoline-3,5′-[1′,2′,4′]-oxadiazol]-2-one derivatives against *S*. *epidermidis*, *S*. *aureus*, *E*. *coli* and *K*. *pneumoniae* using Broth microdilution method [106]. During in vitro studies **27** (Table 3) emerged as the most active derivative with the MIC value of 64 μg/mL against *S*. *epidermidis* exhibiting comparable activity to reference compounds—chloramphenicol and ciprofloxacin and far better than ampicillin. Moreover, the introduction of halogen atoms (chlorine or iodine) into the 5-position of indole ring increased antibacterial activity towards *S*. *aureus*. Regrettably, all of the tested compounds exhibited significantly lower activity than levofloxacin (used as a reference).

Recently, Shetnev A. and collaborators discovered novel 1,2,4-oxadiazole-2-imidazole hybrids as analogs of new class of efflux pump inhibitors presented by Haynes K. M. et al. [107,114]. Unfortunately, during the in vivo test, the instability of amide moiety of the aforementioned efflux pump inhibitors has been observed. For that reason, it was postulated that the replacement of amide moiety with 1,2,4-oxadiazole might lead to an increase of hydrolysis resistance. Antimicrobial activities of novel compounds were evaluated against Gram-positive bacteria *S*. *aureus* and *B*. *subtilis* as well as Gram-negative bacteria *E*. *coli* and *P*. *fluorescent*. Compound **28** (Table 3) emerged as the most potent derivative with the MIC values in a range from 8 to 16 μg/mL (the MIC values for reference compound—pefloxacin—ranged from 0.008 to 0.5 μg/mL). SAR studies revealed that the introduction of alkyl chains into the structure of terminal aromatic ring or the removal of chlorine atoms were related with a decrease of activity. Unfortunately, the influence of other halogen atoms was not evaluated. It is worth emphasizing that **28** exhibited activity against the human pancreas (PANC-1) cancer cell line, leading to growth inhibition up to 80% in a dose-dependent manner. The same research group presented a study of the biological evaluation of 3,5-disubstituted-1,2,4-oxadiazoles containing vinyl moiety in their structure, although the most active compound **29** (Table 3) showed a few hundred-fold lower activity than pefloxacin and fluconazole—used as reference compounds [108].

In 2019, Upare A. A. and coworkers reported the synthesis and biological evaluation of novel 1,2,4-oxadiazole derivatives inspired by the structure of cinnamic acid as antitubercular agents [109]. It has been proved that cinnamic acid and its derivatives exhibited good biological activity against *Mycobacterium tuberculosis*, thus introducing 1,2,4-oxadiazole moiety into the cinnamic acid seemed reasonable in order to improve antitubercular properties [115]. Obtained compounds were examined against *M*. *tuberculosis* (H37Ra). Outcomes indicated that compound **30** (Table 3) exhibited the highest antitubercular activity with the IC_50_ value of 0.045 μg/mL, higher than cinnamic acid itself (IC_50_ = 0.06 μg/mL), however 25-fold times lower than reference compounds—isoniazid and rifampicin (IC_50_ = 0.0019 and 0.0018 μg/mL, respectively). For that reason structural modification to improve biological activity is still required.

Recently, 21 new substituted 1,2,4-oxadiazol-3-ylmethyl-piperazin-1-ylquinolone derivatives have been synthesized as a potent agent against *M*. *tuberculosis*. In vitro evaluation against H37Rv strain, revealed that analog **31** (Table 3) was the most potent and exhibited the MIC value of 0.5 μg/mL, however, isoniazid and rifampicin turned out to be slightly more active (MIC = 0.015 and 0.03 μg/mL, respectively) [110]. Also, compound **31** showed high oral bioavailability and elimination time thus it represents a potent framework for further development as an antitubercular drug.

In 2016, dos Santos Filho J. M. and collaborators synthesized and examined the biological activity of a novel series of 1,2,4-oxadiazole-*N*-acylhydrazone-fused derivatives as potent antimalarial drugs [111]. Biological screening against chloroquine-resistant W2 strain of blood-stage *Plasmodium falciparum* identified compound **32** (Table 3) as the most potent derivative capable of inhibiting growth of microorganisms up to 72% at 10 μg/mL. Moreover, 1,2,4-oxadiazole-*N*-acylhydrazone derivatives exhibited anti-*Trypanosoma cruzi* activity [116,117]. Further in vitro evaluation revealed that **32** demonstrated values of IC_50_ against *P*. *falciparum* (0.02 μM) and CC_50_ against HepG2 (16.9 μM) similar to those determined for reference compound (mefloquine). Despite potent in vitro activity, in vivo experiments failed because none of the mice survived the test of infection with *Plasmodium berghei* (NK65 strains) and no parasitemia reduction has been observed within 30 days of treatment with **32** at 100 mg/kg/day dose. Thus, it seems that 1,2,4-oxadiazole-*N*-acylhydrazones are not suitable for treating malaria, however modification of their chemical structure at both aromatic rings may lead to enhancement of in vivo activity.

Kim J. et al. described 3-aryl-1,2,4-oxadiazole derivatives with human rhinovirus (hRV) activity. Their previous work led to the identification of antiviral compound based on isopropyl benzo[*b*]tiophene-2-carboxylate-derivative exhibiting excellent efficacy against hRVA and hRVB. This study highlighted that metabolic stabilities of tested compounds were unsatisfactory, due to the hydrolysis of ester moiety, thus its replacement with 1,2,4-oxadiazole core have been performed [112,118]. Consequently, a number of 3-aryl-1,2,4-oxadiazole-based derivatives has been synthesized and evaluated against three different human rhinoviruses by cytopathic effect reduction assay. Compound **33** (Table 3) turned out to be the most potent among the series with the IC_50_ values of 66.0, 22.0 and 3.7 nM against hRV-B14, hRV-A21, and hRV-A71, respectively. The reference compound—pleconaril—was less active (IC_50_ = 92.0, 73.0 and 94.0 nM against the same panel of rhinoviruses). Additionally, **33** demonstrated low systemic clearance, moderate oral bioavailability and long half-time in Sprague-Dawley male rats, hence it is an interesting candidate for the development of new antiviral lead compounds.

## 6. Anti-Inflammatory Agents

Inflammation is a complex and natural biological response of body tissues to the injuries and infections. Its function is based on initial cell injury elimination, clearance of necrotic cells or damaged tissues of the body and speeds repair up. Nevertheless, the presence of uncontrolled inflammation may lead to diverse diseases including inflammatory bowel disease, diabetic neuropathy, tumor initiation and progression, osteoarthritis and rheumatoid [119,120,121]. Non-Steroidal Anti-Inflammatory Drugs (NSAIDs)—the most frequently used pain relievers and anti-inflammatory agents—are inhibitors of cyclooxygenases COX-1 and COX-2 (enzymes crucial for the inflammatory process). While COX-1 is produced by kidneys and gastrointestinal tract and its inhibition may lead to many side effects, COX-2 is generated directly during the inflammation process, which is important from the clinical point of view. Nowadays, many traditional NSAIDs, including naproxen, ibuprofen, diclofenac, and aspirin, are non-selective COX inhibitors and many side effects are the consequence of their application. For that reason, the development of new, selective COX-inhibitors is presently ongoing.

Recently, Yatam S. et al. reported synthesis, in vitro and in vivo evaluation of 2-mercapto-benzothiazole-linked 1,2,4-oxadiazoles as potent inflammatory agents [122]. Among obtained derivatives compound **34** (Table 4) occurred to be the most active and selective against COX-2 (the IC_50_ value of 5.0 μM), however, its activity was far weaker than reference compounds—indomethacin and celecoxib—commonly used NSAIDs (the IC_50_ values of 0.36 and 0.038 μM, respectively). Interestingly, in vivo studies of **34** proved its higher activity than that of ibuprofen in carrageenan-induced rat paw edema assay (81% of inflammation inhibition for **34** and 72% of inhibition for ibuprofen, 3h after carrageenan injection).

The same research group disclosed biological activity of benzoxazole derivatives containing 1,2,4-oxadiazole heterocycle as COX inhibitors [123]. Analog **35** (Table 4) exhibited the highest selectivity and activity in in vitro assay (the IC_50_ value against COX-2 is 4.83 μM), but still weaker than reference compounds (indomethacin and celecoxib, the IC_50_ values of 13 and 0.34 μM, respectively). However, in vivo activity of **35** in carrageenan-induced rat paw edema assay was higher than that of ibuprofen (85% and 64% of inhibition for **35** and ibuprofen, respectively, 5h after injection). It is also worth emphasizing that the above-mentioned derivatives showed antioxidant properties in DPPH radical antioxidant assay.

In 2018, a series of 1,2,4-oxadiazol-sulfonamide derivatives was synthesized as selective COX-2 inhibitors [124]. Obtained compounds were tested in vivo in carrageenan-induced rat paw edema assay. Additionally, hot plate and tail immersion methods on rats have been performed. Compound **36** (Table 4) showed the highest anti-inflammatory (55% inhibition of acute inflammation, 3 h after injection at 40 mg/kg single dose) and analgesic activity (5.7 to 14.3 and 4.5 to 8.0 s in hot plate and tail immersion assay, respectively, at a single dose of 40 mg/kg). Unfortunately, the activity of **36** was lower than those observed for reference compounds—aspirin (6.7 to 23.2 and 4.5 to 11.3 s in hot plate and tail immersion assay, respectively, at dose of 10 mg/kg) and indomethacin (71% of inhibition of inflammation, 3h after injection at dose 10 mg/kg), thus improving the activity by structure modification is required.

## 7. Anti-Allodynic Agents

Neuropathic pain is a serious worldwide problem. Nowadays, anti-depressants based on tricyclic structure, anticonvulsants and opioids have been used for chronic pain treatment. Nonetheless, some of them are not effective in all cases, and may cause sever undesirable side effects (even life-threatening addiction and abuses) during long-term treatment [130,131]. Recently, sigma receptors (σ_1_ and σ_2_), initially improperly recognized as opioid receptors (though still their function is not completely understood), have been identified as potential targets in the treatment of central nervous system (CNS) disorders and drug-resistant tumors [132,133].

**Figure 12:**
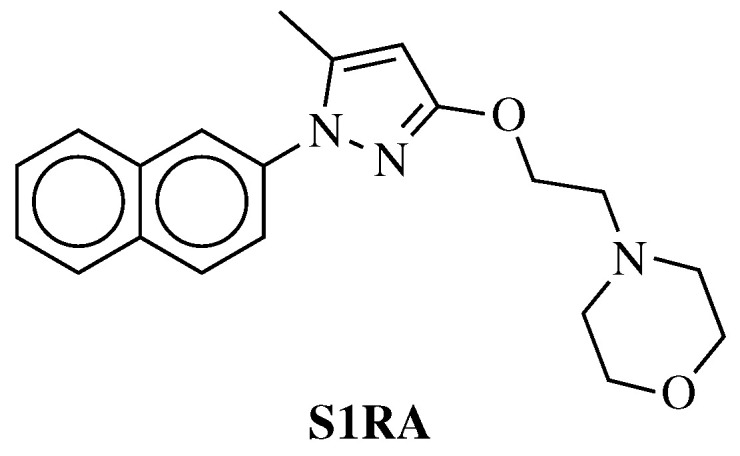
Chemical structure of **S1RA**.

In 2018, Cao X. et al. synthesized and evaluated a series of 3-phenyl-1,2,4-oxadiazole derivatives as potent anti-allodynic agents possessing affinity to σ_1_ and σ_1_ receptors with poor activity to other CNS receptors at the same time [125]. Based on their previous study, the synthesis of hybrids of compounds based on 1,2,4-oxadiazole framework with six-membered heterocyclic rings of pyrimidine and pyridazinone as pharmacophore resulted in improvement of activity [134,135]. Synthesized compounds were evaluated in vitro in primary σ_1_ and σ_2_ binding assay using radiolabelled ligands [^3^H]-(+)-pentazocine and [^3^H]-di-*o*-tolylguanidine, respectively. 3-(2,4-Dichlorophenyl)-5-(4-(piperidin-1-yl)butyl)-1,2,4-oxadiazole **37** (Table 4) showed the highest affinity and selectivity to σ_1_ receptor with K_i_ values of 0.28 nM and 164 nM for σ_1_ and σ_2_, respectively. Surprisingly, **37** activity was higher than reference compound S1RA (Figure 12)—σ_1_ and σ_2_ agonist, currently entered into phase II clinical trials (11, and >2000 nM for σ_1_ and σ_2_, respectively). Additionally, SAR studies revealed that hydrophobic pharmacophore as well as the presence of halogen atoms in the structure of phenyl ring, were crucial for maintaining high biological activity and selectivity. Furthermore, the replacement of chlorine atoms with other halogens or exchanging the piperidine heterocycle drastically decreased activity. Moreover, in vivo studies of **37** in rat formalin test and Chronic Constriction Injury (CCI) pain model assay proved its astonishing potential as a drug against neuropathic pain with good safety profile (LD_50_ = 957 mg/kg). Thus, **37** seems to be an ideal candidate for further in vivo and clinical evaluation, however, no information has been published up to date.

## 8. Anticonvulsant Agents

Epilepsy is a neurological disorder characterized by frequent and unpredictable seizures and affects over 50 million people of all ages worldwide. Unfortunately, the cause of epilepsy occurrence is still unknown, although some incidents are the results of a stroke, brain injury, tumors, infections, or birth defects [136,137]. Nowadays, there are many examples of market-available drugs (including carbamazepine, phenobarbital, phenytoin, diazepam, etc.), nevertheless, approximately for 30% of patients those drugs are ineffective and the occurrence of some undesirable side effects such as dizziness, somnolence and gastrointestinal problems have been observed [138]. For that reason, the development of new, safe and effective anti-epilepsy agents is necessary.

Recently, Mohammadi-Khanaposhtani M. and coworkers presented a number of acridone- and coumarin-based 1,2,4-oxadiazoles, which were tested against pentylenetetrazole (PTZ)- and maximal electroshock (MES)-induced seizures in mice as potent anticonvulsant agents [126,127]. Examined compounds based on acridone as well as coumarin derivatives showed promising anti-epilepsy properties in PTZ and MES assays (with exception of coumarin derivatives in MES assay). It turned out that compound **38** (Table 4) (the ED_50_ values of 2.08 and 3.71 mg/kg in PTZ and MES, respectively) and **39** (Table 4) (100% of seizures protection in mice at 7 mg/kg dose in MES test) showed the highest anti-seizure activity. However, anticonvulsant potency was lower than that of diazepam as a reference (0.68 and 0.98 mg/kg in PTZ and MES assay, respectively, and 100% of seizures protection at 2 mg/kg dose in MES test). For that reason, structural modification (e.g., substitution of the aromatic ring) may show unrevealed features of acridone- and coumarin-fused 1,2,4-oxadiazoles in further development.

## 9. Anti-Alzheimer Agents

Alzheimer’s disease (AD) is a chronic neurodegenerative disease that usually slowly and continuously worsens over time leading to dementia, language-disorders, disorientation, mood swings and behavioral issues, resulting usually in death within 3 to 9 years after diagnosis. Importantly, all over the world AD impacts more than 40 million people leading to death of approximately 2 million people every year. Although, over 100 years have passed since the first AD case has been described, to date the cause of this disease is still poorly understood [139]. Acetylcholinesterase (AChE) and butyrylcholinoesteraze (BChE) are enzymes responsible for the hydrolysis of neurotransmitter in brain tissues—acetylcholine (ACh)—leading to a decrease of its concentration, which is characteristic feature of AD [140]. Nowadays, AChE inhibitors such as galantamine, donepezil, and rivastigmine are used for treating AD, however, their application leads only to a slowdown in the disease development or reduction of AD symptoms, but the progress cannot be stopped or reversed. Therefore, the development of new, effective treatment methods is of special significance.

Recently, Zhang J. et al. performed the synthesis and biological evaluation of coumarin-1,2,4-oxadiazole-fused hybrids as selective BChE and AChE antagonists with potent neuroprotective activity [128]. The previous study of *Phidianidine B* modifications led to the discovery of neuroprotectants against Aβ_25-35_-induced neurotoxicity in human neuroblastoma (SH-SY5Y) cancer cell line [141,142]. Obtained 1,2,4-oxadiazole-coumarin derivatives were evaluated against AChE and BChE. All tested compounds exhibited moderate activity toward AChE with the IC_50_ values ranging from 89.7 to 45.6 μM. Compound **40** (Table 4) turned out to be the most selective BChE inhibitor exhibiting the IC_50_ values of 8.2 and 77.6 μM against BChE and AChE, respectively. Interestingly, the second enantiomer of **40** showed similar activity (IC_50_ = 9.6 and 72.5 μM against BChE and AChE, respectively). Moreover, compound **40** demonstrated significant neuroprotective activity against Aβ_25-35_-induced neurotoxicity in SH-SY5Y cell line (18.8% cell viability increases at 1 μM, compared with Aβ_25-35_ treated cells). Discovering the 1,2,4-oxadiazole/coumarin derivatives may lead to a new molecular framework for developing dual-AChE-BChE inhibitors as anti-Alzheimer agents.

## 10. Anti-Insomnia Agents

**Figure 13:**
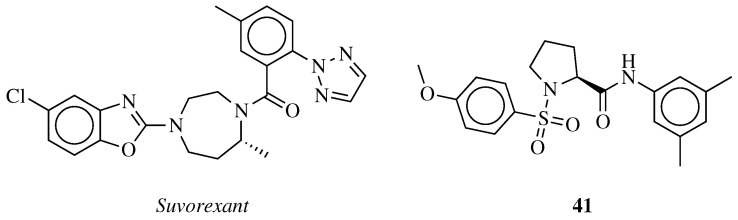
Chemical structures of *Suvorexant* and clinically tested **41.**

Insomnia is a health disruption associated with unsatisfactory or insufficient length of sleep which usually results in a lack of rest, concentration, and ability to learn, bad mood, irritability and sometimes may even lead to cardiovascular diseases, hypertension, dementia or depression. It is estimated that insomnia affects up to 70% of the general adult population making it an enormous public health problem [143]. For many years insomnia was predominantly treated with GABA antagonists, however, high risk of addiction and reduced next-day frame of mind encouraged further development of new anti-insomnia agents. In 1998 the discovery of orexin A and orexin B neuropeptides took place, and since then its antagonists, e.g., almorexant, lemborexant have reached clinical trials [144,145,146,147]. In 2014 the FDA agency approved *Suvorexant* (Figure 13) as the first Dual-Orexin Receptor Antagonist (DORA) for the insomnia treatment sold under the brand name *Belsomra* [148]. However, next-morning somnolence, muscle weakness, weird dreams, sleepwalking are common side effects, thus more potent compounds with better pharmacological profile and safety are still of demand [149].

In the recent study by Brotschi C. and Boss C. the development of new 1,2,4-oxadiazole derivatives as DORAs has been performed [129]. This work is a continuation of the considerable research in the discovery of a potent drug for primary insomnia treatment. In addition, compound **41** (Figure 13), obtained by the above-mentioned research group, entered phase I clinical trials [150,151,152].

Structural hybrids of *Suvorexant* and previously reported piperidine-containing orexin antagonist have been used as starting scaffold [151]. Extensive SAR studies led to the discovery of **42** (Table 4)—an extremely potent DORA, exhibiting the IC_50_ values of 28 and 4 nM against orexin receptors 1 and 2, respectively in FLIPR^®^ calcium release assay. In vivo study on freely moving male Wistar rats revealed that **42**, when administrated *per os* (P.O.) at 100 mg/kg significantly decreased the time spent in active-wake and increased the time spent in non-REM and REM sleep (−24%, +14.3%, and +35.2%, respectively) when compared to vehicle. Moreover, obtained results indicated that compound **42** was more effective than *Suvorexant* (time spent in active-weak, non-REM and REM, −17%, +21.6%, and +21.6%, respectively, when compared with the vehicle). Interestingly, **42** showed analogous results at a lower dose of 30 mg/kg. In summary, further development of 1,2,4-oxadiazole-based DORA agents hold great promise for the discovery of new potent anti-insomnia drugs.

## 11. Other Biological Activities

Kappa-Opioid Receptors play a pivotal role in modulation of dopamine, serotonin and glutamate release in CNS. Recent studies have suggested the KOR involvement in diverse neuropsychiatric or neurological disorders, e.g., epilepsy, addictions, alcohol abuse, depression, schizophrenia and anxiety, therefore the development of novel, efficient KOR antagonist with high selectivity and medication-like profile attracted the medicinal chemists’ interest [153,154,155].

**Figure 14:**
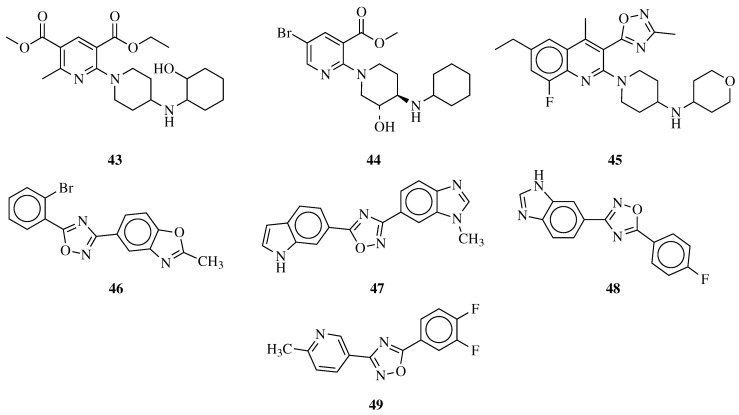
Chemical structures of KOR inhibitors **43**–**45** and Nrf2-ARE activators **46**–**49**.

In 2019, Guerrero et al. discovered novel and selective KOR inhibitors demonstrating potential application in migraine and stress-related mood disorders (e.g., anxiety, depression and drug abuse) treatment [156]. High-throughput screening campaign of the Molecular Libraries-Small Molecule Repository led to identification of hit compound **43** (Figure 14) exhibiting moderate KOR inhibition (the IC_50_ value of 410 nM) with low selectivity against other opioid receptors. Modifications of **43** led to the discovery of highly potent **44** (Figure 14) demonstrating greater selectivity and inhibitory properties [157]. Regrettably, **44** inhibited CYP2D6 and sodium channel site 2 at 10 μM, which increases the probability of cardiovascular liabilities. For that reason, further structural modifications have been made and according to the extensive SAR studies, the selectivity and pharmacological properties were optimized, leading to discovery of the most promising derivative **45** (Figure 14). Analog **45** exhibited high activity against KOR (the IC_50_ value of 0.8 nM) and selectivity over Mu-Opioid Receptor (MOR) (the IC_50_ value of 110 nM) and Delta-Opioid Receptor (DOR) (the IC_50_ value of 6500 nM) [156]. Additionally, **45** was examined against a broad panel of 500 off-targets (inducing kinases, ion channels and other receptors or transporters) and no hits were identified. In vivo pharmacokinetic studies revealed good tissue-distribution and high plasma clearance (105 mL/min kg) after 1 mg/kg single-dose. Encouraged by such promising results, Guerrero and coworkers directed compound **45** into phase I clinical trials for the treatment of neuropsychiatric disorders.

Nuclear Factor Erythroid 2-related factor (Nrf2) signaling pathway plays a crucial role in cells protecting from exogenous and endogenous stresses, e.g., oxidants, xenobiotics, carcinogens and excessive nutrient/metabolite supply. It has been proved that activation of Nrf2 defense response showed protective activity against neurodegenerative diseases (e.g., Alzheimer’s disease), aging, photo-oxidative stress, inflammation, pulmonary fibrosis, pulmonary injury, cardiovascular disease and cancer [158,159,160,161]. Therefore, Nrf2 signaling pathway is an attractive therapeutic target for chemoprevention and chemotherapy drug development as well as for discovery of agents preventing from chronic and neurodegenerative diseases.

In 2015, Xu L. et al. discovered novel, 1,2,4-oxadiazole-based derivatives as active Nrf2 activators—potent anti-inflammatory agents, which is a continuation of their previous work in Nrf2/ARE (Antioxidant Responsive Element) pathway activators development [162,163,164]. Preliminary screening of 7500 in-house compound collection via ARE-luciferase reporter assay using HepG2-ARE-C8 cells revealed moderate Nrf2 activator **46** (Figure 14). However, subsequent molecular similarity search from Chemdiv collection using 2D molecular fingerprint FCFC_6 method and Discovery Studio 3.0 for 3D shape-based similarity search identified **47** (Figure 14) with higher activity (12.41-fold increased ARE level at 40 μM when compared with vehicle sample in luciferase reporter assay). Compound **47** demonstrated no cell toxicity against HCT-116 cell line with dose-dependently proliferative inductivity at remarkably high concentration of 40 μM. Moreover, in vitro studies revealed that **47** was able to maximize the expression of several phase II antioxidant enzymes (HO-1, NQO1) and to enhance Nrf2 expression in a dose-dependent manner. Further structure modification based on SAR studies led to the discovery of **48** (Figure 14) exhibiting the most promising ARE inductivity and physicochemical properties, and therefore, it has been chosen as a lead compound for detailed evaluation [162]. In summary, **48** showed dose-dependent induction of the expression level of Nrf2 in *q*RT-PCR and Western-blot analysis. Moreover, in vivo studies in C57BL/6 female mice showed a great reduction of proinflammatory cytokines with no bodyweight affection after **48** administration.

The same research group developed novel derivatives of **48** applicable in the treatment of liver diseases. Broad structural modification emerged novel, lead compound **49** (Figure 14) possessing enhanced ARE-inducing activity and more favorable physicochemical properties when compared with previously evaluated **48** [165]. It has been proved that **49** promoted nuclear translocation of Nrf2 and increased its expression in normal liver cells L02 without hepatotoxicity. In addition, hepatocytes-protecting properties have been observed in vivo in APAP-induced acute liver damage. It is also worth emphasizing that **49** showed an ideal therapeutic effect on MPTP-induced Parkinson’s disease in mice, improving behavioral abnormalities and reducing chemically induced dopaminergic neuron loss and secretion of inflammatory factors [166]. Summarizing, Xu L. et al. discovered multipotent drugs which are able to activate the Nrf2 pathway with potential application in the treatment of neurodegenerative diseases and APAP-induced liver injury.

## 12. Conclusions

1,2,4-Oxadiazole nucleus and its derivatives seem to be an auspicious framework in the discovery and development of drugs exhibiting immense bioactivities. It has been revealed from foregoing considerations that several 1,2,4-oxadiazole-based compounds may have significant importance in the synthesis of novel agents potentially useful in the treatment of cancer, inflammation, insomnia, Alzheimer’s disease, and abuses or addictions. Some of compounds described in this paper are suitable for clinical studies and their evaluation is still ongoing, holding great promise for the development of novel drugs. Moreover, one of them has recently entered into phase I of clinical trials.

Recently, the proteolysis-targeting chimeras (PROTACs) strategy, based on bifunctional molecules designed to recruit an E3 ubiquitin ligase to a specific target protein, has become very popular. Recent reports indicate that the E3 complex and target protein facilitates the processive transfer of ubiquitin from the E3 complex to the target protein, thereby tagging the pathological protein for degradation via the proteasome [167]. To date, only a few bifunctional compounds have been successfully designed based on PROTACs technology. Considering the universality of oxadiazole based compounds, their broad spectrum of biological activities as well as simplicity of chemical modifications, their application in such technologies should be invaluable in the near future.

The ever-growing interest in this class of compounds is forcing scientists to develop new, efficient and environmentally friendly methods of synthesis. One of the latest synthetic approaches is the application of mechanochemistry. These techniques (grinding or milling) are a powerful strategy for the rapid, clean, and solvent-free synthesis of many biologically active compounds [168]. These reactions are usually performed in a mixer ball mill or mortar grinder and are of great value due to the possibility of reducing or completely eliminating the use of solvents, enhancing the conversion of substrates or even obtaining products that were unavailable with the previously used methods [169]. In addition, in many cases, the use of the above techniques allows for a significant reduction of reaction time and saving of synthesis costs. In the future the synthetic strategy may contribute in obtaining many new drug candidates, including very promising derivatives based on 1,2,4-oxadiazole scaffold.

## Figures and Tables

**Table 1:** Methods of synthesis of 1,2,4-oxadiazole derivatives.

	Starting Material	Reagents	Substituents	Conditions	Products	Advantages and Limitations	Ref.
Entry 1	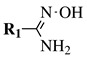	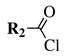	**R_1_**, **R_2_** = methyl or phenyl.	Solvent-free; Melting.	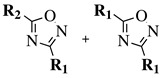	Low yields; Long reaction time; Difficult purification; Presence of by-products.	[11]
Entry 2	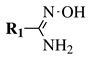	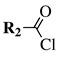	**R_1_** = phenyl, 2-methylphenyl, 3-methylphenyl, 4-methylphenyl, 4-nitrophenyl or methyl; **R_2_** = methyl, phenyl, 2-nitrophenyl, 3-nitrophenyl, 4-nitrophenyl, benzyl, methoxy, chloromethyl, *t*-butyl or trifluoromethyl.	THF; RT; TBAF as catalyst.	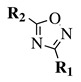	Short to long reaction time (1–72 h); RT; Poor to excellent yields (<5% to 98%).	[30]
Entry 3	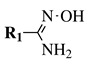	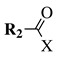	**X** = methoxy, ethoxy or Cl; **R_1_** = methyl, chloromethyl, phenyl or 4-methylphenyl; **R_2_** = benzyl, 4-methylbenzyl, 4-methoxybenzyl or *t*-butylpropionate.	Toluene; Reflux; K_2_CO_3_.	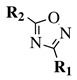	Good yields (50–95% for esters and 70–79% for acyl chlorides); Moderate to short reaction time (∼12 h for esters and ∼2 h for acyl chlorides); Easy work-up.	[31,32]
Entry 4	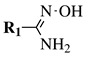	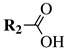	**R_1_** = H, *t*-butyl, 4-*t*-butylphenyl, 2,4-dichlorophenyl, 4-bromophenyl, 4-methyl-1,2,3-thiadiazol-5-yl or 3-methyl-4-nitrophenyl; **R_2_** = 4-bromophenyl, *N*-Boc-azetidine-3-yl,1-naphthyl, *N*-Boc-aminoethyl or 5-methyl-3-thiophen-2-yl.	TEA; T3P; ∼80 °C.	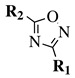	Excellent yields (87–97%); Short to moderate reaction time (0.5–6 h); Easy work-up; Expensive activating agent (T3P).	[34]
Entry 5	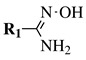	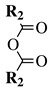	**R_1_** = phenyl, 4-chlorophenyl, 4-bromophenyl, 2,4-dichlorophenyl, 4-methoxybenzyl or cyclohexyl; **R_2_** = phenyl, 4-methylphenyl or *n*-pentyl.	H_2_O; Reflux; 12 h.	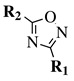	Low to excellent yields (35–93%); Moderate reaction time (12 h); Catalyst-free; Organic solvent-free; Aqueous medium.	[35]
Entry 6	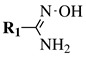	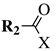	**X** = hydroxy, methoxy, ethoxy or Cl; **R_1_** = phenyl, *o*-, *m*-, *p*-tolyl, 4-chlorophenyl, 3-bromophenyl, 4-bromophenyl, 4-nitrophenyl or 4-methoxyphenyl; **R_2_** = 3-oxo-1-butyl, phenyl, 2,4-dichlorophenyl, 4-chlorobenzyl or cyclohexyl.	Solvent-free; NH_4_F/Al_2_O_4_ as a catalyst; MWI; ∼10 min.	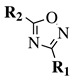	Moderate to excellent yields (40–90%); Remarkably short reaction time (∼10 min); Organic solvent-free; Simply work-up; Absence of by-products.	[36,37]
Entry 7	**R_1_**-CN	**Step I**: NH_2_OH·HCl MWI; **Step II**: **R_2_**COCl or **R_2_**CHO; MWI.	**R_1_** = phenyl, 4-chlorophenyl, 4-bromophenyl, 4-methylphenyl, and many others (see Ref.); **R_2_** = phenyl, 4-methoxyphenyl, 3-nitrophenyl, and many others (see Ref).	**Step I**: Solvent-free; CH_3_COOH, MgO or Na_2_CO_3_ as catalyst; MWI; **Step II**: Solvent-free; MWI.	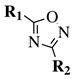	Usually excellent yields (>90%); One-pot procedure; Cheap catalysts; Remarkably short reaction time (∼2–10 min); Organic-solvent-free; None by-products; Easy work-up.	[37,38,39,40]
Entry 8	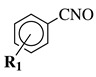	**R_1_**-CN	**R_1_** = 2,4,6-trimethyl or 2,4,6-trimethoxy; **R_2_** = methyl or ethyl.	**Step I**: [PtCl_4_-R’_2_(CN)]_2_ (R’ = CH_3_, CH_3_CH_2_, PhCH_2_), CH_3_CN, CH_2_Cl_2_; **Step II**: Pyridine, CH_2_Cl_2_.	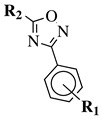	Low yields; Long reaction time (up to 72 h); Poor solubility of Pt compounds; Difficult purification.	[43]

**Table 2:** 1,2,4-oxadiazole derivatives with antitumor activity.

General Structure	Substituents	The Most Active Derivatives	Activity	Ref.
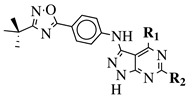	**R**_1_ = H, NH_2_ and other (see Ref.); **R**_2_ = H or phenyl.	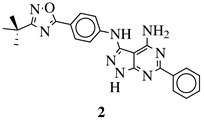	IC_50_ values of 2.76 and 9.27 μM against OVXF 899 and PXF 1752 cancer cell lines, respectively.	[55]
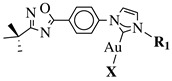	**X** = Cl or Br; **R_1_** = methyl, benzyl, 2-pyridinyl or anthracen-9-ylmethyl.	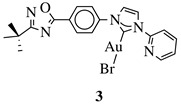	IC_50_ values of 3 nM against LXFA 629 and MAXF 401 cancer cell lines, respectively.	[56]
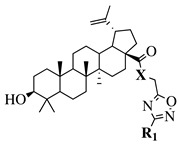	**X** = O or NH; **R_1_** = phenyl, benzyl, 2-chlorophenyl, 4-fluorophenyl, 2-mehylphenyl, 4-bromophenyl, 4-methylphenyl, 4-methoxyphenyl, 4-pyridinyl, 2-methoxyphenyl, 2-benzyloxyphenyl or 3-pyridinyl.	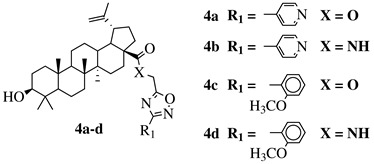	IC_50_ values between 26.1–34.3 μM against Colo 205, Hep G2 and Hela cell lines.	[57,58]
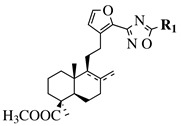	**R_1_** = methyl, chloromethyl or phenyl.	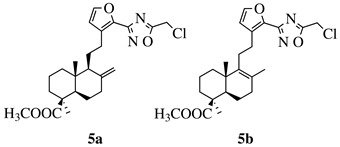	GI_50_ values of 0.08 (**5a**) and 0.34 (**5b**) μM against CEM-13 cell line.	[60]
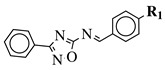	**R_1_** = H, Br, Cl, F, methoxy or NH_2_.	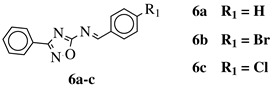	CC_50_ values of 137.3, 79.0 and 140.3 μM against Ca9-22 cell line, respectively.	[63]
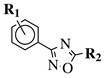	**R_1_** = H, 2-chloro, 3-chloro, 4-chloro, 4-nitro, 4-methyl, 4-methoxy, 4-trifluoromethyl, 2-bromo, 3-bromo, 4-bromo or 4-fluoro; **R_2_** = N(CH_3_)_2_, N(C_2_H_5_)_2_, pyrrolidine-1-yl, azepan-1-yl, morpholin-1-yl, thiomorpholine-1-yl, *N*-methylpiperazin-1-yl, *N*-phenylpiperazin-1-yl, 3-bromopropan-1-yl or 3-chloropropan-1-yl.	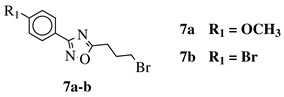	80% of death of NB4, K562 and MDA-MB-231 cancer cell lines at 25 (**7a**) and 10 (**7b**) μM.	[64]
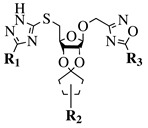	**R_1_** = H or NH_2_; **R_2_** = isopropylidene or cyclopentylidene; **R_3_** = 4-nitrophenyl, 4-chlorophenyl or 3,4,5-trimethylphenyl.	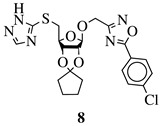	GI_50_ of 4.5 μM against WiDr cancer cell line.	[65]
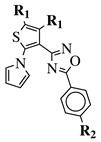	**R_1_** = CH_3_ or —(CH_2_)_4_—; **R_2_** = H, Cl, Br, methyl or methoxy.	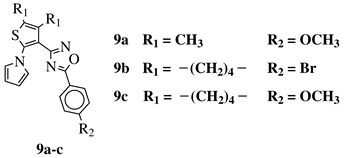	IC_50_ values of 0.48 (**9a**), 0.78 (**9b**), 0.19 (**9c**) μM against MCF-7 cancer cell line.	[66]
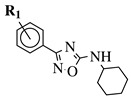	**R_1_** = H, 3-methyl, 4-methyl, 3-bromo, 4-methoxy, 4-trifluoromethyl, 4-chloro, 4-bromo or 4-fluoro.	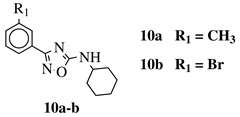	IC_50_ values between 13.6–48.37 μM against HCT-116, PC-3, SNB-19, B16F10, L929 cell lines.	[67]
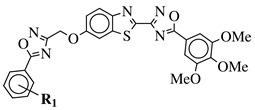	**R_1_** = H, 3,4,5-trimethoxy, 4-methoxy, 4-chloro, 4-bromo, 4-fluoro, 4-trifluoromethyl, 4-nitro, 4-cyano or 4-methyl.	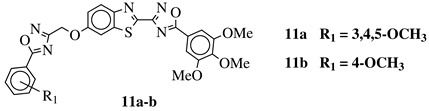	IC_50_ values in a range from 0.11 to 2.09 μM against MCF-7, A375 and HT-29 cancer cell lines.	[68]
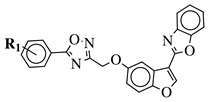	**R_1_** = H, 3,4,5-trimethoxy, 4-methoxy, 4-chloro, 4-bromo, 4-fluoro, 4-trifluoromethyl, 4-nitro, 4-cyano or 4-methyl.	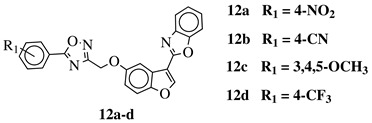	IC_50_ values between 0.011–1.89 μM against A549, MCF-7, A375 and HT-29 cancer cell lines.	[69]
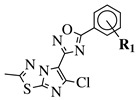	**R_1_** = H, 3,4,5-trimethoxy, 4-methoxy, 4-chloro, 4-bromo, 4-fluoro, 4-trifluoromethyl, 4-nitro, 3-nitro or 4-methyl.	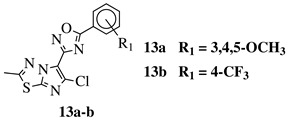	IC_50_ values in a range of 0.11–1.47 μM against A375, MCF-7 and ACHN cancer cell lines.	[70]
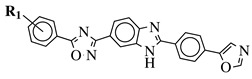	**R_1_** = H, 3,4,5-trimethoxy, 4-methoxy, 4-chloro, 4-bromo, 4-fluoro, 4-trifluoromethyl, 4-nitro, 4-cyano or 4-methyl.	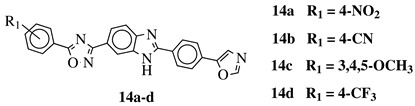	IC_50_ values between 0.12–2.78 μM against MCF-7, A549 and A375 cancer cell lines.	[71]
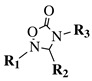	**R_1_** = methyl, phenyl, 4-fluorophenyl, benzyl or 4-methoxbenzyl; **R_2_** = phenyl, 9-phenanthryl or 4-pyridinyl; **R_3_** = 4-nitrophenyl, 4-chlorophenyl, 4-trifluoromethylphenyl or 4-fluorophenyl.	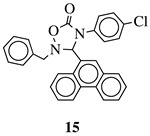	IC_50_ value of 10.38 μM toward MCF-7 cancer cell line.	[72]
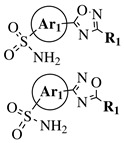	**R_1_** = methyl, phenyl, 2-methoxyphenyl, 3-methoxyphenyl, 4-*t*-butylphenyl, 4-methylphenyl, 2-methoxyphenyl, 3-methoxyphenyl, cyclopropyl, 2-pyridinyl, 3-pyridinyl, 4-pyridinyl, 2-thienyl, 3-thienyl, 4-cyanophenyl, 2-fluorophenyl, 3-fluorophenyl, 4-fluorophenyl, 2-chlorophenyl or 3,4-dichlorophenyl; **Ar_1_** = *p*-phenylene, *m*-phenylene, *p*-methoxyphenylene or 2,4-thienyl.	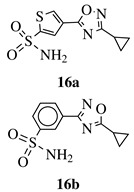	K_i_ value of 89 pm and 0.75 nm (hCA IX and hCA II, respectively) for **16a** in CO_2_ hydration stopped-flow biochemical assay. **16b** showed high selectivity toward PANC-1 cancer cell line.	[73,74]
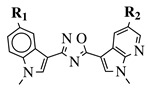	**R_1_** = H, F, Cl, Br or methoxy; **R_2_** = H, F or Br.	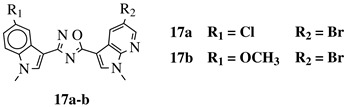	IC_50_ values of 0.65 (**17a**) and 2.41 μM (**17b**) against MCF-7 cancer cell line.	[75]
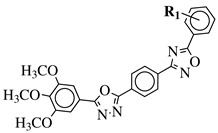	**R_1_** = H, 3,4,5-trimethoxy, 4-methoxy, 4-chloro, 4-bromo, 4-fluoro, 4-nitro, 3-nitro, 4-cyano or 4-trifluoromethyl.	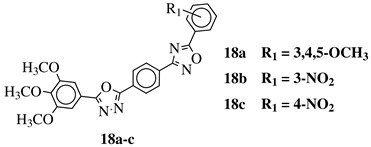	IC_50_ values in a range of 0.45–2.11 μM against MCF-7, A549, MDA-MB-231 cancer cell lines.	[76]
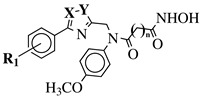	**X**, **Y** = N, O or O, N; **n** = 5 or 6; **R_1_** = H, 2-methyl, 4-methyl, 4-methoxy, 2-fluoro, 3-fluoro, 4-fluoro, 4-bromo or 4-nitro.	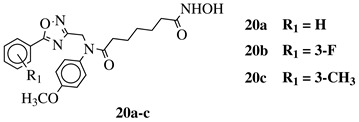	IC_50_ values of 8.2, 10.5, 12.1 nM (**20a**, **20b**, **20c**, respectively) toward HDAC-1.	[77,78]
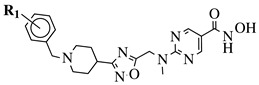	**R_1_** = H, 4-methyl, 3-methyl, 2-fluoro, 4-fluoro, 2,4-difluoro, 2-chloro, 4-cyano, 4-trifluoromethyl or 2-chloro-4-fluoro.	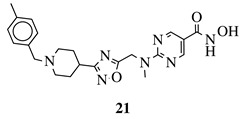	IC_50_ values of 1.8, 3.6 and 3.0 nM against HDAC-1, -2 and -3, respectively.	[79]
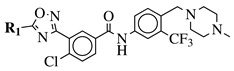	**R_1_** = 3-pyridinyl, 4-pyridinyl, 4-methoxy-3-pyridinyl, 5-(2-methoxyethoxy)-3-pyridinyl, 5-morpholin-3-pyridinyl or 5-(1-methyl-1*H*-pyrazol-3-yl)-3-pyridinyl.	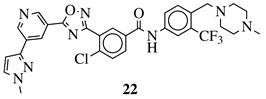	IC_50_ value of 7.3 nM against RET enzyme in ELISA assay.	[80]

**Table 3:** 1,2,4-oxadiazole derivatives and their antimicrobial activity.

General Structure	Substituents	The Most Active Derivatives	Activity	Ref.
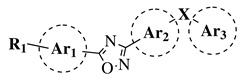	**R_1_** = H, OH, OCH_3_, NH_2_, NHAc, NH_3_Cl, NHMs, NH-*n*Bu, NH-*t*Bu, NHCOPh, NH-*i*Pr, PO_3_H_2_, PO(OEt)_2_, SO_2_NH_2_, CONH_2_, COOH, COOCH_3_ F, Cl, Br, I, NO_2_, ethynyl or CN; **Ar_1_** = phenyl, benzyl, 2-pyrole, 3-pyridyl, 4-pyridyl, 5-indole, 3-pyrrazole, 2-imidazole and many others (see Ref.); **Ar_2_**, **Ar_3_** = *p*-phenylene, 6-indole, 2-pyridyl, 6-chromene, carbazole, *N*-phenylpiperazine, *N*-phenylmorpholine and many others (see Ref.); **X** = NH, CH_2_, O, CO, NBn, SO or SO_2_.	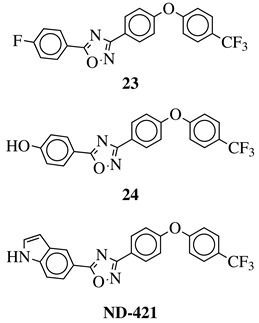	MIC_50_ values <4 μg/mL against over 210 diverse, MRSA and VRE strains.	[96,98,99,103]
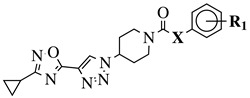	**X** = NH or none; **R_1_** = H, 3-chloro-4-fluorophenyl, 2-chlorophenyl, 2-ethyl, 4-ethyl, 5-bromo-2-fluorophenyl or 2-methylpyridin-5-yl.	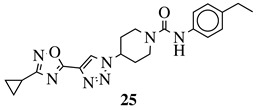	Grown inhibition zone within 20–25 mm against *S*. *aureus*, *B*. *subtilis*, *E*. *coli*, *P*. *vulgaris*, *P*. *aeruginosa*, *C*. *albicans*.	[104]
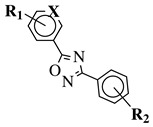	**R_1_** = H, 2-chloro or 3-chloro; **X** = CH or N; **R_2_** = H, 2-nitro, 2-chloro, 3-bromo, 2-chloro-5-nitro, 2-bromo, 3-nitro, 2-iodo, 3,5-dinitro, 4-nitro or 2-hydroxy.	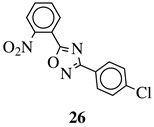	MIC value of 60 μM against *E*. *coli*.	[105]
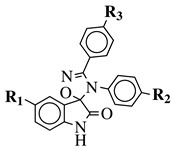	**R_1_** = H, F, Cl, Br, I, methyl, ethyl, methoxy or *i*Pr; **R_2_** = H, methyl, methoxy, *i*Pr, F, Cl, Br or I; **R_3_** = H, F, Cl, Br, nitro, *i*Pr, OBn, methoxy, ethoxy or CN.	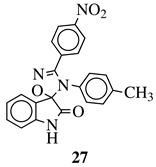	MIC value of 64 μg/mL against *S*. *epidermidis*.	[106]
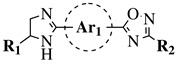	**R_1_** = H or methyl; **Ar_1_** = *p*-phenylene or *m*-phenylene; **R_2_** = methyl, cyclopropyl, 2-thienyl, 2-chlorophenyl, 3-chlorophenyl, 3,4-dichlorophenyl, 4-ethylphenyl, 4-*t*-butylphenyl, 4-methylphenyl, 3,4,-dimethylphenyl and many others (see Ref.).	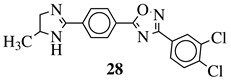	MIC values in a range 8–16 μg/mL toward *S*. *aureus*, *B*. *subtilis*, *E*. *coli*, *P*. *fluorescent*.	[107]
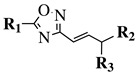	**R_1_** = phenyl, 4-mehtoxyphenyl, 4-chlorophenyl, 3-methylthienyl or 2-pyridinyl; **R_2_**, **R_3_** = H, methyl, phenyl, 4-chlorophenyl, 4-methoxyphenyl, 3,4,-dimethoxyphenyl or 2,3-dimethoxyphenyl.	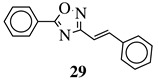	MIC value of 0.68 mM against *S*. *aureus*.	[108]
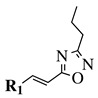	**R_1_** = phenyl, 4-methylphenyl, 4-methoxyphenyl, 4-methylthiophenyl, 2-chlorophenyl, 4-chlorophenyl, 2-3-dichlorophenyl, 3,4-dichlorophenyl, 4-fluorophenyl, 4-bromophenyl, 4-hydroksyphenyl, 2-bromo-4-fluorophenyl, 4-cyanophenyl, 4-pyridinyl, 1-napthyl and others (see Ref.).	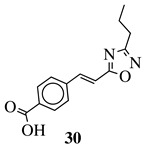	IC_50_ value of 0.045 μg/mL against *M*. *tuberculosis* (H37Ra).	[109]
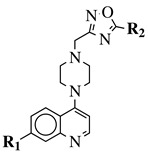	**R_1_** = 4-pyridyl, 3-pyridinyl or 3,5-difluorophenyl; **R_2_** = 3,5-dimethoxyphenyl, 3,5-difluorophenyl, 3-cyanophenyl, 2,3-dimethylphenyl, cyclopentyl or 4-izopropylphenyl.	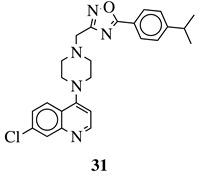	MIC value of 0.5 μg/mL against *M*. *tuberculosis* (H37Ra).	[110]
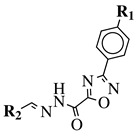	**R_1_** = H, F, Cl, Br, methyl, nitro, methoxy or hydroxy; **R_2_** = 4-hydroksy-3-methoxyphenyl, 2-styryl, ferrocene or 5-benzo[1,3]dioxole.	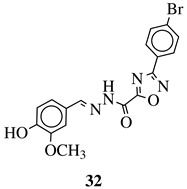	IC_50_ value of 0.02 μM against *P*. *falciparum*. In vivo studies failed—none in vivo activity.	[111]
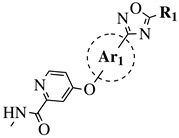	**R_1_** = Me, Et, cyclopropyl, *i*Pr, CF_3_, *i*Bu or CH_2_OCH_3_; **Ar_1_** = *p*-phenylene, *p*-2-methylphenylene, *p*-2,6-dimethylphenylene, 2,5-pyridinyl or 3-methylbenzothiophene	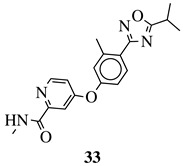	IC_50_ values of 66.0, 22.0 and 3.7 nM against hRV-B14, hRV-A21 and hRV-A71, respectively.	[112]

**Table 4:** 1,2,4-oxadiazole derivatives and their antimicrobial activity.

General Structure	Substituents	The Most Active Derivatives	Activity	Ref.
**ANTI-INFLAMMATORY ACTIVITY**				
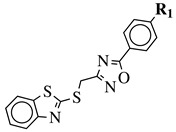	**R_1_** = NO_2_, CF_3_, F, Cl, COOH, COOCH_3_, CON(CH_3_)OCH_3_, CONH(*t*-Bu) and others (see Ref.).	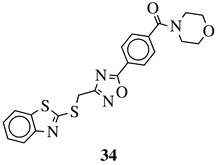	IC_50_ value of 5.0 μM against COX-2.	[122]
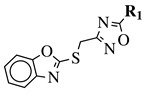	**R_1_** = 2-fluorophenyl, 4-fluorophenyl, 2,4-difluorophenyl, 2,5-difluorophenyl, 2-chlorophenyl, 3-chlorophenyl, 4-chlorophenyl, 2,4-dichlorophenyl, 4-nitrophenyl, 4-trifluoromethylphenyl, 4-methylphenyl, 3-pyridinyl or 5-thiazole.	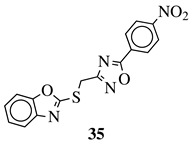	IC_50_ value of 4.83 μM against COX-2.	[123]
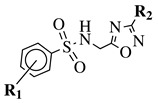	**R_1_** = H, 4-methyl, 4-methoxy or 4-chloro; **R_2_** = 4-fluorophenyl, 4-methylphenyl, 4-chloro, 4-methoxyphenyl or 2-chlorophenyl.	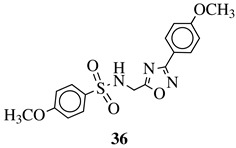	55% inhibition of acute inflammation (3 h after injection at 40 mg/kg).	[124]
**ANTI-ALLODYNIC ACTIVITY**				
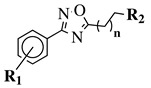	**R_1_** = 2-napthyl, 4-methylphenyl, 4-chlorophenyl, 4-fluorophenyl, 2,3-dichlorophenyl, 2,4-dichlorophenyl, 2,5-dichlorophenyl, 3,5-dichlorophenyl, 3,4-dichlorophenyl, 3-chloro-4-fluorophenyl or 3,4-difluorophenyl; **n** = 2 or 3; **R_2_** = *N*-morpholine, *N*-piperidine, 4-methyl-*N*-piperdine, 3,5-dimethyl-*N*-piperidine, *N*-piperidin-4-one, *N*-methyl-*N*-piperazine, *N*-ethyl-*N*-piperazine, *N*-pyrrolidine, *N*-dimethylamine or *N*-diethylamine.	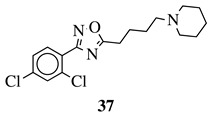	K_i_ values of 0.28 nM and 164 nM for (σ_1_ and σ_2_, respectively).	[125]
**ANTICONVULSANT ACTIVITY**				
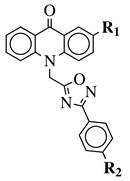	**R_1_** = H, Cl, methoxy, Br, methyl or ethyl; **R_2_** = methyl, Cl or methoxy.	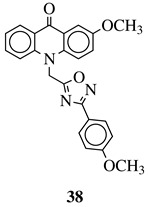	ED_50_ values of 2.08 and 3.71 mg/kg in PTZ and MES, respectively.	[126]
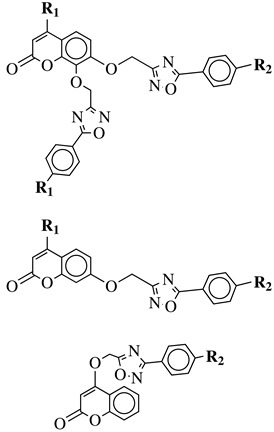	**R_1_** = H, methyl or phenyl; **R_2_** = methyl, Cl or Br.	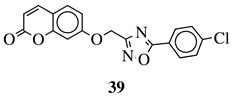	100% of seizures protection (in an assay with mice at 7 mg/kg dose in a MES test).	[127]
**ANTI-ALZHEIMER ACTIVITY**				
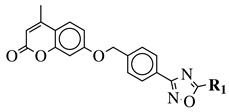	**R_1_** = phenyl, 2-methylphenyl, 3-methylphenyl, 4-methylphenyl, 3-methoxyphenyl, 4-methoxyphenyl, 4-*N*,*N*-diethylaniline, 4-ethynylphenyl, 2-fluorophenyl, 3-fluorophenyl, 4-fluorophenyl, 4-chlorophenyl, 4-bromophenyl, 2-nitrphenyl, 3-nitrophenyl, 4-nitrophenyl and others (see Ref.).	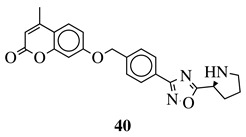	IC_50_ values of 8.2 and 77.6 μM against BChE and AChE, respectively.	[128]
**ANTI-INSOMNIA ACTIVITY**				
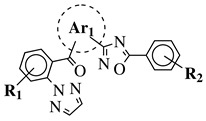	**R_1_** = H, Cl, methyl, F or methoxy; **R_2_** = H, methyl, methoxy, Cl, F or OCF_3_; **Ar_1_** = piperazine, diethylamine, ethylamine, pyrrolidine or azetidine and others (see Ref.).	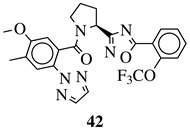	Decreases the time spent in active-wake and increases time spent in non-REM and REM sleep (−24%, +14.3% and +35.2%, respectively, at 100 mg/kg).	[129]

## Abbreviations

The following abbreviations are used in this manuscript:

AChE	Acetylcholinesterase
AD	Alzheimer Disease
AIDS	Acquired Immunodeficiency Syndrome
APAP	Acetaminophen
AMF	Acute Myeloid Leukemia
ARE	Antioxidant Responsive Element
BChE	Butyrylcholinoesterase
CCI	Chronic Constriction Injury
CDI	1,1′-Carbonyldiimidazole
CNS	Central Nervous System
COX	Cyclooxygenase
CXCR4	Chemokine Receptor Type 4
DCC	*N*,*N*’-Dicyclohexylcarbodiimide
DOR	Delta-Opioid Receptor
DORA	Dual-Orexin Receptor Antagonist
EDC	1-Ethyl-3-(3-dimethylaminopropyl)carbodiimide
EDG	Electron Donating Group
ELISA	Enzyme-Linked Immunosorbent Assay
ER	Estrogen Receptor
Et	Ethyl
EWG	Electron Withdrawing Group
FDA	Food and Drug Administration
GABA	*gamma*-Aminobutyric Acid
*h*CA	Human Carbonic Anhydrase
HDAC	Human Deacetylase
HDSirt2	Human Deacetylase Sirtuin 2
HEDMs	High Energy Density Materials
HIV	Human Immunodeficiency Virus
hRV	Human Rhinovirus
KOR	Kappa-Opioid Receptor
Me	Methyl
MES	Maximal Electroshock
MOR	Mu-Opioid Receptor
MPTP	1-Methyl-4-phenyl-1,2,3,6-tetrahydropyridine
MRSA	Methicillin-Resistant *Staphylococcus aureus*
MWI	Microwave Irradiation
NAD^+^	Oxidized Nicotinamide Adenine Dinucleotide
Nrf2	Nuclear Factor Erythroid 2
NSAIDs	NON-Steroidal Anti-Inflammatory Drugs
PBP2	Penicillin-Binding Protein 2
Ph	Phenyl
P.O.	*per os*
PROTACs	Proteolysis-targeting chimeras
PTP1B	Protein-Tyrosine Phosphate 1B
PTZ	Pentylenetetrazole
RT	Room Temperature
REM	Rapid-Eye Movement
RET	Rearranged During Transfection
SAR	Structural-Activity Relationship
T3P	Propylphosphonic anhydride
TBTU	2-(1*H*-Benzotriazole-1-yl)-1,1,3,3-tetramethylaminiumtetrafluoroborate
TBAF	Tetra-*n*-butylammonium fluoride
TEA	Triethylamine
TfOH	Trifluoromethanesulfonic acid
THF	Tetrahydrofuran
VRE	Vancomycin-Resistant *Enterococcus faecium*
VRSA	Vancomycin-Resistant *Staphylococcus aureus*
